# *Aspergillus terreus* (Trichocomaceae): A Natural, Eco-Friendly Mycoinsecticide for Control of Malaria, Filariasis, Dengue Vectors and Its Toxicity Assessment Against an Aquatic Model Organism *Artemia nauplii*

**DOI:** 10.3389/fphar.2018.01355

**Published:** 2018-11-26

**Authors:** C. Ragavendran, R. Srinivasan, Myunghee Kim, Devarajan Natarajan

**Affiliations:** ^1^Natural Drug Research Laboratory, Department of Biotechnology, School of Biosciences, Periyar University, Salem, India; ^2^Department of Food Science and Technology, College of Life and Applied Sciences, Yeungnam University, Gyeongsan, South Korea

**Keywords:** *Aspergillus terreus*, neurobehavioral toxicity, knock-down effects, histopathology, mosquito coil, smoke toxicity, *Artemia nauplii*

## Abstract

Vector-borne diseases like malaria, filariasis, and dengue are transmitted by mosquitoes and they cause global mortality and morbidity due to an increased resistance against commercial insecticides. The present study was aimed to evaluate the neurobehavioral toxicity, knock-down effect, histopathology, ovicidal, adulticidal, and smoke toxicity effect of *Aspergillus terreus* extract against three mosquito species, namely *Anopheles stephensi, Culex quinquefasciatus*, and *Aedes aegypti* (Diptera: Culicidae). The isolated fungal strain was identified as *A. terreus* (GenBank accession no: KX694148.1) through morphological and molecular (phylogenetic) analysis. The morphological changes in the treated fourth instar larvae shown the demelanization of cuticle and shrinkage of the internal cuticle of anal papillae. The time duration of extract exposure against the larvae determines the level of toxicity. The extract treated larvae were displayed excitation, violent vertical and horizontal movements with aggressive anal biting behavior as the toxic effect on the neuromuscular system. The results of the biochemical analysis indicated that a decrease in the level of acetylcholinesterase, α-carboxylesterase, and β-carboxylesterase in extract treated fourth instar larvae of all tested mosquito species. The findings of histopathological investigation shown the disorganization of the abdominal region, mainly in mid, hindgut, and gastric caeca, loss of antenna, lateral hair, caudal hair, upper and lower head hairs in the mycelium extract treated *An. stephensi, Cx. quinquefasciatus*, and *Ae. aegypti*. The ovicidal bioassay test results showed the mosquito hatchability percentage was directly related to the concentrations of mycelium extract. Nil hatchability of mosquito eggs was noticed at 500 μg/ml concentration. The adulticidal activity of fungal mycelia ethyl acetate extract resulted in a dose-dependent activity (15 and 30 min recovery periods). The higher concentration of extract (1000 mg/L) acted as a repellent, the adult mosquitoes showed restless movement, uncontrolled/anesthetic flight at last died. The better adulticidal activity was observed in the ethyl acetate extract against *An. stephensi, Cx. quinquefasciatus* followed by *Ae. aegypti* with the best score of LD_50_ and LD_90_ values and nil mortality was found in the control. The results of smoke toxicity assay of the mycelia extract exhibited significant mortality rate against *Ae. aegypti* (91%), *Cx. quinquefasciatus* (89%), and *An. stephensi* (84%). In addition, the present investigation reported the stability and toxic effects of *A. terreus* mycelium ethyl acetate extract on *Artemia nauplii*. The swimming speed (0.88 mm s^-1^) of *A. terreus* was reduced with ethyl extract 24 h treatment whereas, the control *A. nauplii* showed the normal speed of 2.96 mm s^-1^. Altered behavior and swimming movement were observed in the 8 h *A. terreus* mycelium extract treated *A. nauplii*. A pale yellow color substance (metabolites) was found in the mid-gut region of the mycelial extract exposed *A. nauplii*. The outcome of the present study, suggest that the *A. terreus* metabolites might serve as an alternative, cost-effective, eco-friendly, and target specific mosquitocidal agent in the future.

## Introduction

Mosquitoes serve as medically important/significant vectors, they can transmit parasites and pathogens to the humans which leads to the devastating impact on human health. Three million people deaths every year due to malaria (Snow et al., 2005; Benelli and Mehlhorn, 2016) and the mortality is the main causes of infant and young child (WHO, 2000). *Culex quinquefasciatus* is a most irritating vectors and important man-biting mosquitoes leads to cause allergic responses, including local skin and systemic diseases, including angioedema and urticaria (Cheng et al., 2008; Vadivalagan et al., 2017). It works as a vector for lymphatic filariasis, Japanese encephalitis and West Nile virus (JE) in India (Gopalakrishnan et al., 2014; Dev et al., 2015). *Wuchereria bancrofti* cause lymphatic filariasis and *Cx. quinquefasciatus* transmit this disease and found to be endemic to India, it can infect above 465 million individuals worldwide/annually (WHO, 2011).

Dengue is one of the most significant mosquito borne disease to spread various viral diseases and play an important role in public health concern. It also occurred in the world, especially tropical and subtropical regions and most prevalence in urban and semi-urban areas. *Aedes aegypti* is a vector of several important arbovirus, with special reference to dengue and Zika virus causing yellow fever, chikungunya, and dengue in other severe forms, causes dengue hemorrhagic fever and dengue shock syndrome (Patil et al., 2012; Benelli and Romano, 2017). DF or DHF is caused by dengue virus which includes different serotypes 1, 2, 3, and 4 (Den-1, Den-2, Den-3, and Den-4) (WHO, 2010). In recent times, the incidence of dengue has enlarged severely all over the world and about 80 million public infected by dengue, and reported 4% global attack rate/year (Caraballo and King, 2014). Recently, dengue infections have been significantly raised due to extensive urbanization, intensive trade and travel. So far, there is no effective drug (or) vaccine is available. To avoid the breeding of disease carrying mosquito and biting humans is the only solution for mosquito control.

Currently, the use of organochlorine, organophosphate, and synthetic pyrethroid insecticides for control of mosquitoes by public health sprays. Successive changes in the insecticides resulted multiple insecticides resistant in malaria and other vectors. In India, malarial vectors are resistant to several insecticides like DDT, HCH, malathion, and deltamethrin (Raghavendra et al., 2010). The problem of insecticide resistance has led to the search for alternative vector control measures, including a biological way of mosquito control. The application of microbial insecticides is preferred due to exhibit selective toxicity, do not persist after application, are relatively safer to produce and possess fewer (or) less toxic to non-target organisms and environmental hazards (Misato, 1983).

Endophytic or entomopathogenic microbes and their conidia act as potential biological control agents. Therefore, these soil fungi and their metabolites may provide an environmentally safe and effective control of mosquito. *Aspergillus terreus* is a filamentous ascomycete, commonly found in soil. It develop aerial hyphae and unique among *Aspergilli* in producing lateral cells termed aleurospores in the absence of typical conidiophore structures in submerged culture (Klich and Pitt, 1992). The use of *A. terreus* isolates in the fermentation industry for the production of itaconic acid, protease enzyme (Bigelis and Arora, 1992; Lowe, 1992; Osman et al., 2014), antibiotics, antifungal, antibacterial, and antineoplastics (Rizna et al., 2008); sterriquinone, butyrolactone I, cytrinin, lovastatin, and terrecyclic acid (Schimmel et al., 1998; Vertesy et al., 2000). The recent research on *A. terreus*, produce some metabolites namely mevinolinic acid, (+)-geodin, asterric acid, and butyrolactone I (Boruta and Bizukojc, 2016), patulin, citrinin, and gliotoxin (GT), which are harmful to humans (Pastor and Guarro, 2014). The other beneficial compounds like terrrein (a malanogenesis inhibitor), asperfuranone, and cyclosporine A (an immune-suppressant drug) (Guo and Wang, 2014; Yin et al., 2016) have been produced from *A. terreus*. Besides, citric acid, gluconic acid, itaconic acid, and kojic acid are commercially produced from *A. terreus* (Lv et al., 2014; van der Straat et al., 2014). Aspulvinone, an active natural product capable of inhibiting influenza An H1N1 virus (Gao et al., 2013). In *A. terreus* used genome-mining studies that have identified 10 genes are linked with the biosynthesis of terretonin (Guo et al., 2012). Generally, the pigments produced by *Aspergillus* sp. has antibacterial, antifungal activity (Teixeria et al., 2012), antiangiogenesis (Dikmen et al., 2017) and also used in the textile dyeing (Atalla et al., 2011), degrade the hydrocarbon (Vatsyayan and Goswami, 2016), phytotoxic and herbicidal activities (Khattak et al., 2014). *A. terreus* acts as a biocontrol agent against *Biomphalaria alexandrina* snails (Saad et al., 2014), and promoter for plant growth, and adverse effects of stem rot disease caused by *Sclerotium rolfsii* (Waqas et al., 2015). The toxicity of the soil-borne fungal extracts from *Aspergillus* species (*A. flavus, A. niger*, and *A. parasiticus*) (Maurya et al., 2011), *Beauveria tenella* (Balaraman et al., 1979), *B. bassiana* (Ragavendran et al., 2017), *Lagenidium giganteum* (Lacey et al., 1988; Vyas et al., 2007), and *Chrysosporium lobatum* (Mohanty and Prakash, 2008) are widely reported as against mosquito larvae. Earlier, Ravindranath and Kapadnis (1991) investigated the isolation of *Fusarium oxysporum* and *Fusarium pallidoroseum* from mosquito larvae, and test their potential against stages of mosquito larvae.

Acetylcholinesterase (AChE) enzyme is sometimes called as true or specific cholinesterase and mainly found in nerve cells, skeletal, smooth muscle, various glands, and red blood cells in animals/insects (Ellman et al., 1961; Hawkes et al., 2005; Colovic et al., 2013). This may be differentiated from other cholinesterases by substrate and inhibitor specificities. These types of mechanism (ex. organophosphate and carbamate chemicals) affect the transmission of nerve impulses accumulating acetylcholine in the neuromuscular tissue of insects causing paralysis and finally death (Planche et al., 2012). Therefore, the discovery of insect AChE inhibitors is an important task (Casanova et al., 2002; Liu et al., 2012) and it is generally accepted the physiological role of AChE by the rapid hydrolysis and inactivation of acetylcholine.

Carboxylesterases are ubiquitous non-specific enzymes it hydrolyse a variety of esters of carboxylic acids and play a major role in insect metabolic activity, i.e., regulation of juvenile hormone (JH), fat metabolism and mobilization, energy-related fat catabolism in muscles, cuticular wax synthesis and transport (Satoh and Hosokawa, 2006; Wheelock et al., 2008) mechanism for organophosphate resistance in insects (Hemingway and Karunaratne, 1998) and metabolism of exogenous insecticidal substrates (Ross et al., 2010), respectively.

*Artemia* sp. used as a nutritious live food source for a variety of marine organisms (Radhika Rajasree et al., 2010), and characterized by the common features viz: short life cycle, high adaptability to adverse environmental conditions, small size, higher offspring production, and easy to culture (Nunes et al., 2006). *Artemia* is identified as best species by the US Environmental Protection Agency (EPA, 2002) for acute and eco-toxicity tests (Persoone and Wells, 1987; Nunes et al., 2006; Kokkali et al., 2011). Hence, the toxicity of several chemical compounds (including pesticides and antifouling biocides) has been tested using *Artemia* sp., in the last few decades (Venkateswara Rao et al., 2007; Garaventa et al., 2010; Alyuruk et al., 2013). Recently, eco-toxicological tests were done by Ates et al. (2013) using the *Artemia* sp. to assess the hazard of different bioactive metabolites/nanoparticles effects on the marine environment.

With the brief introduction, the present study focused on the neurobehavioral toxicity, knock-down effects, histopathology, ovicidal, adulticidal, and smoke toxicity of *A. terreus* extract against mosquito vectors *Anopheles stephensi, Cx. quinquefasciatus*, and *Ae. aegypti* and also check its bio-toxicity against a model organism *Artemia nauplii*.

## Materials and Methods

### Materials and Reagents Used

The following necessary chemicals/media used in this study, i.e., acetylcholinesterase (AChE), acetylthiocholine iodide (AChEI), fast blue B, sodium dodecyl sulfate (SDS), 5,5-dithiobis-2-nitrobenzoic acid (DTNB), α- and β- naphthyl acetate and fungal growth medium, potato dextrose agar (PDA), potato dextrose broth (PDB), and sabouraud dextrose broth (SDB) were procured from Hi-Media, Mumbai. The chemicals like ethyl acetate, dimethyl sulfoxide (10% DMSO) and others were obtained from Merck (Germany) and Sigma-Aldrich, United States.

### Isolation and DNA Extraction From *A. terreus*

The isolation and identification (morphology and microscopy) steps of *A. terreus* from soils has been reported by Ragavendran and Natarajan (2015). The isolated fungal strain was cultured in PDA medium for 7 days at 25°C (Abreu et al., 2003). Mycelial biomass were extracted for DNA analysis using the modified DNA isolation procedure (Sambrook et al., 1989; Passone et al., 2010). The mycelium of fungal isolate (100 mg aliquot) was transferred into 1.5 ml of centrifuge tubes. The mycelium was vortexed for 1 min in the addition of 300 μl sterile water plus 700 μl of extraction buffer (100 mM Tris–HCl, 2% CTAB, 1.4 mM NaCl) and glass beads (425–600 μm diameters) for the disruption of fungal mat material. After incubation at 65°C for 60 min, 500 μl of chloroform was added to the sample, homogenized and centrifuged at 10,000 rpm (for 10 min). The aqueous phase was collected and add 500 μl of chloroform. Again, the sample was homogenized and centrifuged at 10,000 rpm (for 10 min). The aqueous phase was recovered and precipitated with 2 volumes of precipitation buffer (14 mM CTAB, 40 mM NaCl and pH 8.0). After incubated at room temperature for 1 h, the sample was homogenized and centrifuged at 10,000 rpm (for 5 min). Then, the sample was homogenized by inversion, in the occurrence of NaCl (1 M) and chloroform (each 350 μl) and centrifuged at 10,000 rpm (for 5 min). The recovered chloroform phase was precipitated by adding 0.6 ml of isopropanol (at -20°C). After incubation at room temperature (for 20 min), it was centrifuged at 10,000 rpm (for 10 min) and the aqueous phase was discarded. Finally, the DNA pellet was washed with 70% ethanol and suspended in 50 μl of nuclease-free H_2_O. Polymerase chain reaction (PCR; TECHNE TC-512, Barloworld Scientific Ltd., United Kingdom) was performed in 50 μl reactions with the following concentrations: 5 U μl^-1^ of Invitrogen (Brazil) Taq DNA polymerase, 5X Invitrogen Buffer, 2 mM of dNTPs, 1.5 mM Mg^2+^, and 3 pmol μl^-1^ of each primer. The 5.8S rDNA (5′–GRAAGNAHADGTVGKAAYAWSG–3′) ITS primers (5′–TCCTNCGYTKATKGVTADGH–3′) (O’Donnell et al., 1998) were manufactured by Invitrogen Custom Primers (Carlsbad, CA, United States). PCR was run by adopting the following steps: initial denaturation at 94°C for 1 min, followed by 31 cycles of denaturation at 94°C for 30 s, annealing at 56°C for 45 s, extension at 72°C for 1 min, and final extension at 72°C for 5 min and the reaction was kept at 4°C. The obtained PCR product was confirmed in the agarose gel electrophoresis.

### Gel Electrophoresis

Agarose gel electrophoresis (DNA) were done based on the modified method of Saghai-Maroof et al. (1984). About 2g of agarose (Bio-Rad agarose, Qiagen) was prepared in 98 ml 1× TAE (Tris/Acetate/EDTA) buffer for produce a 2% solution and heated using a water bath. The solution was allowed to cool at 60°C, prior to the addition of 4 ml ethidium bromide (Et Br) (10 mg/L in water to a final concentration) and thoroughly mixed. The gel was poured in glass plates. Polymerase chain reaction products (Iheanacho et al., 2014) were slowly loaded (2 ml) into the agarose wells. The current (voltage of 5 V/cm) was passed to the gel for running electrophoresis and PCR product was viewed under Gel documentation system. Then, the molecular size of obtaining DNA was determined by gel electrophoresis and allowed for separation and visualization of DNA fragments from *A. terreus*.

### Purification, Sequencing, and Identification of *A. terreus*

The purification of PCR products was performed using DNA Wizard Clean-Up Kit (product A9282, Promega, Madison, WI, United States). The purified PCR products were compared with Invitrogen’s Low DNA Mass Ladder for estimate the concentration of DNA required for sequencing. Using an Applied Biosystem ABI 3730 sequencer (Applied Biosystem) for sequencing the isolated DNA sample. The PCR product was sequenced in the forward and reverse directions, and consensus sequences were created by using BioEdit program version 7.0.9.0 (Thompson et al., 1994).

### Phylogenetic Analysis

Internal transcribed spacer (ITS) sequences of the *A. terreus* were compared with the available data in NCBI. Multiple sequence alignment was performed with Clustal X software (Thompson et al., 1997), with gaps treated as missing data. The phylogenetic analysis of DNA sequences from *A. terreus* was constructed using the neighbor joining method (Saitou and Nei, 1987) and the Kimura two parameter distance calculation was carried out with the help of mega software version 3.1 (Kumar et al., 2004). The nods of the tree were supported by 1,000 bootstrap replications.

### Collection of Mosquitoes

Larvae of *An. stephensi, Cx. quinquefasciatus*, and *Ae. aegypti* were collected from Centre for Research in Medical Entomology (CRME-ICMR), Madurai and they were maintained in the Natural Drug Research Laboratory, Periyar University, Salem. The mosquitoes were reared in an appropriate equipment, i.e., dechlorinated tap water and maintained at 27 ± 2°C, 70–75% relative humidity (RH), under conditions of 14 h light:10 h dark (LD 14:10) photoperiod cycles without exposure of any pathogens or insecticides. The larvae fed on 3:1 ratio of dog biscuits and yeast powder as a food. Water has been changed every day to avoid scum formation, which may produce toxicity.

### Preparation of Metabolites From *A. terreus*

Extraction of secondary metabolites, larvicidal, and pupicidal activity, FTIR, GCMS, and HPLC analysis of metabolites from *A. terreus* were previously reported by Ragavendran and Natarajan (2015). The fourth instar larvae of tested mosquitoes were collected from pathogen free deionized water. The various concentrations of test metabolite were prepared in 100 ml water for performing bioassay (100, 200, 300, 400, and 500 μg/ml). To check the bioassay of fungal intracellular metabolites, 30 larvae of each stage were separately exposed to 100 ml of test concentrations. Similarly, the each sample along with a negative control (treated with DMSO-distilled water) was tested and the experiment was repeated in five times.

### Larval Mortality and Morphology Study of Three Mosquitos’

A total of 30 fourth instar larvae were separately released in three replicates for each concentration. The cumulative larval mortality was recorded. Larvae was failed to move after probing with a blunt metallic probe in the thoracic segments is considered as dead and the dead larvae were collected for further study. The abnormal shape, size, discoloration, or failures to pupate (larval-pupal intermediates) growth deformations of larvae were characterized. Pupal deformations (abnormal change in shape, size or failure to develop adult), stage (pupal-adult intermediates), and adult malformation (abnormal change in color, shape, and size) of mosquitoes were observed. All survival larvae in treated and control were maintained in the different beakers for further notifications (upto 6 days). The larvae were also monitored carefully for any changes in pigmentation pattern. During an experimental time, brewer’s yeast was not provided to adult mosquitoes (Warikoo and Kumar, 2013).

### Neurobehavioral Toxicity and Knock-Down Effects

The neurobehavioral toxicity effect of ethyl acetate extract of *A. terreus* against mosquito larvae were studied by the modified method of Maketon et al. (2014) and Soonwera and Phasomkusolsil (2016). The behavioral observations of mycelia extract treated and non-treated control groups were monitored (upto 2 h) and photographed by using Nikon D-SLR Digital Camera (Nikon Inc., Japan), equipped with kenko close-up lens. Neurobehavioral toxicity responses of larvae include: impaired, incapability of rising to the water surface, tremors and paralysis was accessed. The knock-down bioassay of extract was performed and the total number of larvae knock down was counted after 2 h of treatment.

### Preparation of Whole Body Homogenates

The fourth instar larvae (both controls and treated) were taken separately and washed with double distilled water and the surface water was removed by blotting with tissue paper. Larvae (40 Nos) were homogenized with 4 ml phosphate buffered saline (PBS) contains a fresh Eppendorf tube. Then, it was homogenate using centrifugation at 8000 rpm for 15 min. After, the clear supernatant was collected and the pellet was discarded. The supernatant was stored on ice (4°C) until it will be used for performing biochemical assays (Sugumar et al., 2014).

### Acetylcholinesterase Assay

The acetylcholinesterase assay of the fourth instar (control and untreated) larval homogenate of selected mosquito was assayed by the method of Ikezawa and Taguchi, (1981) with minor modifications. The stock solutions of test mycelial metabolites were prepared by dissolving DMSO. About 50 μl of homogenate was sequentially mixed with 450 μl of PBS [(100 mM, pH 7.4), 50 μl of 10 mM 5, 5-dithiobis 2-nitro benzoic acid (DTNB)] and 50 μl of 12.5 mM acetylcholine iodide was acting as the substrate. Then it was incubated at room temperature for 5 min and optical density (OD) was measured at 400 nm using Shimadzu spectrophotometer (Kyoto, Japan) including a blank or control reagent (acetylthiocholine iodide).

### Carboxylesterase Assay

The α- and β-carboxylesterase activity of the fourth instar larval homogenates was performed as per the modified method of Van Asperen (1962). In briefly, 200 μl of control and treated fourth instar larval homogenate was incubated along with 2 ml α- or β- naphthyl acetate solution at room temperature (for 30 min). To each reaction mixture, 500 μl of fast blue B and SDS reagent was added [22.5 mg fast blue B salt in 2.25 ml distilled water and 5% w/v SDS in 0.2 M phosphate buffer (pH 7.2)] for arresting the enzymatic reaction. Then, it was allowed to form a color (for 15 min). The optical density (OD) of the sample and respective blank was measured in the Shimadzu spectrophotometer (Kyoto, Japan) (at 588 nm).

### Histopathology Study

The *A. terreus* ethyl acetate extract treated and control larvae (fourth instar) were fixed in paraformaldehyde solution (10%) for 1 week followed by embedded in paraffin. Larval tissue blocks were sectioned (7 μm) using the microtome (Leica, Germany), mounted on the glass slide and stained with haematoxylin and eosin for visualization purpose [under a bright-field microscope (Senthil-Nathan, 2007)]. The sections were observed and photographed using a light microscope (Optika vision lite 2.0 ML) connected with computer. The midgut cells of the treated and untreated larvae of testing mosquitoes were photographed. The actual site of action in the midgut of treated larvae was observed and compared with control. As a control, the larvae were treated with tap water for 24 h.

### Ovicidal Activity

The modified Su and Mulla (1998) method was used for performing the ovicidal activity. The different concentrations of extract (100–500 mg/ml) were prepared from the stock solution. Before treatment, the eggs of *An. stephensi, Cx. quinquefasciatus*, and *Ae. aegypti* were counted under a microscope. Use of freshly hatched eggs of these mosquito species (50 Nos) were exposed to each dose of *A. terreus* ethyl acetate extract. Eggs treated with DMSO in water served as control. After treatment, the egg from each concentration was transferred to distilled water cups for assessing the hatching ability. Each test has replicated in three times. The egg hatches ability rate was assessed after 48 h treatment using the formula as given below.

(1) $$\mathrm{Mortality}\;\mathrm{of}\;\mathrm{egg}(\%)=\frac{\mathrm{Number}\;\mathrm{of}\;\mathrm{hatched}\;\mathrm{larvae}}{\mathrm{Total}\;\mathrm{number}\;\mathrm{of}\;\mathrm{eggs}\;\mathrm{in}\;\mathrm{treated}\;\mathrm{sample}}\times 100$$

### Adulticidal Bioassay

The adulticidal bioassay of extract against tested mosquitoes was performed by the modified WHO (1981) protocol. The known concentrations of the *A. terreus* fungus ethyl acetate extract was dissolved in 2.5 ml of ethyl acetate and poured on clean glass (55 ml) test tube, as per the method described by Dua et al. (2010). The control test tubes also treated with ethyl acetate under similar conditions. The fungal mycelia extract was evaluated at five concentrations (100, 300, 500, 800, and 1,000 mg/L) produced a wide range of mortality from 10 to 100% along with the control. Twenty mosquitoes (2–5 days old, sucrose-fed, blood starved) were collected and gently transferred into a glass holding tube. A pad of cotton, soaked with 10% glucose solution and it was placed on the mesh screen. The mortality of mosquitoes was determined at the end of 30 min/ recovery period. The percent mortality of mosquitoes was corrected using the Abbott’s formula (Abbott, 1925).

$$\mathrm{Percentage}\;\mathrm{mortality}=\frac{\mathrm{Number}\;\mathrm{of}\;\mathrm{dead}}{\mathrm{Number}\;\mathrm{of}\;\mathrm{introduced}}\times 100$$

### Preparation of Mosquito Coil

The mosquito coils were prepared from the extract by the modified method of Saini et al. (1986). Two grams of *A. terreus* mycelium ethyl acetate extract, 1 g of saw dust and 1 g of coconut shell (2:1:1) used as a burning material. All the materials were mixed thoroughly in sterile distilled water to produce a semi-solid paste, and it was used to develop a mosquito coil (with a thickness of 0.5 ± 2.0 cm). The coils were shade-dried and used for further experiments.

### Smoke Toxicity Test

Smoke toxicity test of sample was performed in a glass chamber (measuring 60 cm × 40 cm × 35 cm) with the mid bottom. A total of 50 blood-fed *An. stephensi, Cx*. *quinquefasciatus*, and *Ae. aegypti* adult mosquitoes released into the chamber, and exposed to the smoke from burning coils (for 40 min). The mortality data were recorded at 10 min interval periods (10, 20, 30, and 40 min). The smoke toxicity of fungal based mat was also compared with commercially available mosquito coil (prallethrin) (Singha et al., 2011).

### Biotoxicity Assay of Extracts Against *A. nauplii*

The dehydrated cysts of *A. nauplii* transferred into petri dishes (6 cm) and each dish containing 4 ml of 0.22 μm natural filtered sea water (30% salinity). The incubation was done in a thermostatic room (at 25°C) with a photoperiod of 16 h light and 8 h dark environments (Lubzens et al., 1985). For obtaining stage I larvae, 50 mg of cysts were incubated for 24 h at 28°C under 16 h light, 8 h dark conditions with continuous supply of aeration. After this period, newly hatched *A. nauplii* was separated from non-hatched cysts based on their positive phototaxis and it was transferred using a Pasteur pipette into a glass beaker (10–15 larvae/ml).

### Evaluation of Mortality and Swimming Speed Alteration

The evaluation of mortality and swimming speed alteration of non-target organism (*A. nauplii*) was assessed by the modified methods of Ragavendran et al. (2017) and Benelli et al. (2018). Five different concentrations of extract (100, 200, 300, 400, and 500 μg/ml) were used for this study. The known amount of the stock suspension was immediately transferred to the exposure glass beaker containing *A. nauplii* (*N* = 25/per beaker). The distilled water containing 10% (*v/v*) DMSO served as blank control. The mortality and other abnormalities (such as sluggishness and swimming) of *A. nauplii* was observed. Mortality analysis was done based on the larval activity and the larvae shown completely motionless were counted as dead organisms, and the percentage of mortality was calculated compared to the control. The experimental setup and formula used for measuring swimming speed alteration (SBR system) has been described by Faimali et al. (2006).

(2) Alteration(%)=[(STREATED−SCONTROL)/SCONTROL]*100

The *A. terreus* mycelium ethyl acetate extract was evaluated against *A. nauplii* and LC_50_ were calculated for each concentration using Probit analysis (Finney, 1971). The understanding of post treatment effect of extract on survival and swimming responses of target organism (*A. nauplii*) was observed for 5 days.

### Calculation and Statistical Analysis

The larval knock-down percentage was calculated by the following formula: Larval knockdown (%) = KDL/TL × 100, where KDL corresponds to the number of knock-down larvae and TL to the number of larvae treated. The pupation percentage was carried out using the formula: Pupation (%) = PN/TL × 100, where PN corresponds to the number of pupae formed and TL to the number of larvae treated. The total number of larvae knock-down was subjected to Probit analysis (Finney, 1971) using the SPSS IBM (Statistical Package of Social Sciences, Inc., Chicago, IL, United States) version 20.0 to determine EC_50_ and chi-square values. All the data were expressed as one-way ANOVA performed with Tukey’s honest significant difference (HSD) *P* < 0.05. The percentage of adult mortality data were subjected to Probit analysis for calculate LD_50_, LD_90_, and other statistics like at 95% of the upper confidence limit (UCL) and lower confidence limit (LCL) values and chi-square tests were calculated. Graphs were designed using GraphPad Prism version 5.0 for Windows (GraphPad Software, San Diego, CA, United States).

## Results

The molecular characterization of isolated genomic DNA from *A. terreus* was carried out by PCR amplification. In this method, known universal and species specific primer were used and resulted good specificity for ITS sequence of fungal species. The amplified ITS1-5.8S rDNA-ITS2 regions from the *A. terreus* strain shown PCR products size of 555 bp (GenBank accession number: KX694148.1) and it was sequenced by comparing the ITS sequences of organisms presented in the NCBI GenBank database using a BLAST search tool and MEGA 6 to generate a phylogenetic tree by Neighbor-joining method (Supplementary Figure 1). The optimal tree with the sum of branch length of 1.683 was obtained from database analysis. The highest (99–100%) similarities with the amplified sequences were derived for alignment and boot-strapping using CLUSTAL W, based on the percentage of replicate trees in associated with bootstrap test.

The *A. terreus* mycelium ethyl acetate extract exhibited strong neurobehavioral toxicity for *An. stephensi, Cx. quinquefasciatus*, and *Ae. aegypti* larvae (Supplementary Figures 2a,b). The larvae shown a normal feeding and zigzag wriggling movement (upto l.5–10 min) during the exposure of extract. We clearly observed that larval feeding behavior in the water column and bottom surfaces. This event was disappeared in larvae treated with the extract (at varying concentrations of 200–500 μg/ml). The results expressed an aggressive behavioral symptoms changes in the mosquito viz: impaired coordination, irregular movements, and forceful self-biting (Supplementary Figure 2c), compared to control group. Supplementary Figure 2d illustrate normal behaviors of mosquito within 15 min of treatment. These irregular features and other behavioral symptoms of treated larvae were not stopped and it’s becoming more irritated. After treatment, we noticed the larval up and down, wriggling movements, fail to reach the surface of water (within a limited time period), and several of them having displayed vibration movements (tremors) and paralysis symptoms (Supplementary Figure 2e), but the control showed normal behavioral responses (Supplementary Figure 2f) against extract tested. Overall, the results of lethal study of extract exhibited high levels of knock-down effect against *An. stephensi, Cx. quinquefasciatus*, and *Ae. aegypti* fourth instar larvae (Table 1) with an EC of 2.864, 1.910, and 5.115 μg/ml (after 2 h of treatment). It is observed that the increased knock-down affects corresponds to the concentrations of mycelial extract.

**Table 1:** Knock-down effects of *Aspergillus terreus* mycelium ethyl acetate extract against fourth instar larvae of *Anopheles stephensi, Culex quinquefasciatus*, and *Aedes aegypti* (after 4 h of treatment).

Mosquito species	Extract concentration (μg/ml)	Larval knockdown (%) ±SD^a^	EC_50_ (95% confidence intervals)	χ^2^ (*df* = 3)
*An*. *Stephensi*	100	16.0 ± 0.2e	2.864 (0.000–18.839)	2.172
	200	30.2 ± 0.1d		
	300	45.3 ± 0.0c		
	400	63.1 ± 0.2b		
	500	72.0 ± 0.5a		
	Control	0		
	(DW+DMSO)			
*Cx*. *quinquefasciatus*	100	17.2 ± 0.0e	1.910 (0.000–14.986)	1.930
	200	31.0 ± 0.1d		
	300	44.3 ± 0.2c		
	400	62.1 ± 0.4b		
	500	69.0 ± 0.2a		
	Control	0		
	(DW+DMSO)			
*Ae*. *aegypti*	100	15.0 ± 0.4e	5.115 (0.000–24.304)	3.042
	200	29.2 ± 0.3d		
	300	46.1 ± 0.2c		
	400	64.4 ± 0.3b		
	500	75.0 ± 0.0a		
	Control	0		
	(DW+DMSO)			


*EC_*50*_ values are calculated for five doses (100, 200, 300, 400, and 500 μg/ml) and five replications. 10% DMSO, dimethylsulfoxide; DW, distilled water; EC_*50*_, efficient dose that knock-down 50% of larvae. ^*a*^Means in each row against each mosquito species followed by the difference letters are significantly different (*P* < 0.05, one-way ANOVA followed by Tukey’s test).*

Larval survival and adult emergence of tested mosquitoes were considerably decreased and (fourth instar larvae of *An. stephensi*) exposed to various concentrations of mycelium extract. The control groups achieve 100% survival rate in the entire study periods. A sub-lethal dose of mycelium extract (at 100 μg/ml) induced the growth disruption effects and it was characterized by normal dead larva (Supplementary Figure 3a), failure of larvae molt into pupae, resulted the development of an abnormal larval-pupal intermediates (Supplementary Figure 3b). The deformed pupa (DP) exhibited looking as elephant and it called as “elephantoid” (Supplementary Figure 3c). Deformed pupa (DP) was shown retarded abdomen and wing pads are (WP) not properly developed into the body (Supplementary Figures 3d,e). The aborted adult emergence with legs and wings stuck in pupal caste were noticed (Supplementary Figure 3f).

The results of morphogenetic abnormalities of *Cx. quinquefasciatus* induced by *A. terreus* mycelium ethyl acetate extract and control fourth instar larva were presented in Supplementary Figure 4a. The lethality of higher concentration of mycelium extract shown remarkable and declined the growth of larvae into pupae resulted into unfinished melanization and development of abnormal dead larval-pupal intermediates (Supplementary Figures 4b,c). At sub-lethal doses (100 and 200 μg/ml), we noticed that molting continued in normal but immature stages of mosquito was significantly affected. The microscopic examination of the dead immature stages (at 40× magnifications) revealed the following abnormalities in the mosquito viz: abnormal dead larval-pupal intermediates, deformed pupa (DP) distortion of digestive tract, enlarged cephalothorax, emergent adults with mouthparts and wings folded within the pupal exuvium (Supplementary Figures 4d,e). Few of the emerged adult mosquito unable to escape from the pupal caste and it was dying in the water surface of beaker (Supplementary Figure 4f).

The low concentration (100 μg/ml) of mycelial extract (after 48 h of treatment) acted as moderate level of morphological deformities and growth or molting associated deformities in dead larvae (Supplementary Figure 5) of *Ae. Aegypti*, i.e., deformed feeding brush and head, pigmented cuticles in the thorax and abdomen, shaded/albino colored cuticles in the abdomen, black cure, and pigmented anal papillae, totally disrupted digestive tract system, gut lumen and peritrophic membrane (PM) boundaries (Supplementary Figure 5a). Whereas, the control larvae shown healthy feeding brush and head, healthy cuticles in the thorax and abdomen, intact digestive tract and its boundaries (Supplementary Figure 5b), respectively.

The outcome of results exhibited the growth and molting deformities in the fourth instar larvae of mosquito which include in the formation of larval-pupal intermediates (larviform pupae) and albino colored (white) pupal cuticles in the cephalothoraxes. We noticed the following modification in the treated mosquito namely the anterior and mid region of the abdomen, malformed rudiments of appendages and compound eyes (Supplementary Figure 5e), albino colored pupal cuticles with larval exuviate attached in the cephalothoraxes (Supplementary Figure 5d), pupal cuticles with larval exuviae in the posterior region of the abdomen (Supplementary Figure 5f). We observed pupae have been completely failed to emerge of adults. The exposure of mycelium extract at various concentrations (200–300 μg/ml) induced morphological deformities resulted dead pupae (after 72 h of treatment) viz: black and pigmented cuticles in the cephalothoraxes, the anterior region of the abdomen without larval exuviae (Supplementary Figure 5g), compared to control which shown healthy cuticles in the cephalothorax, abdomen region and normal pupal characteristics (Supplementary Figure 5c).

The overall results observed that only 37% larvae was successfully pupated at 100 μg/ml concentration (after 72 h treatment). Malformation in the developed adults was not clearly noticed, but slightly deformed wings were recorded (Supplementary Figure 5h) after 84 h treatment. The adult emergence was totally stopped in the higher concentrations of 200 and 300 μg/ml due to deformities and death of pupae. While, the 97% pupation rate was observed in control after 72 h treatment and 100% adults emerged in control group (after 120 h treatment) (Supplementary Figure 5i).

**FIGURE 1:**
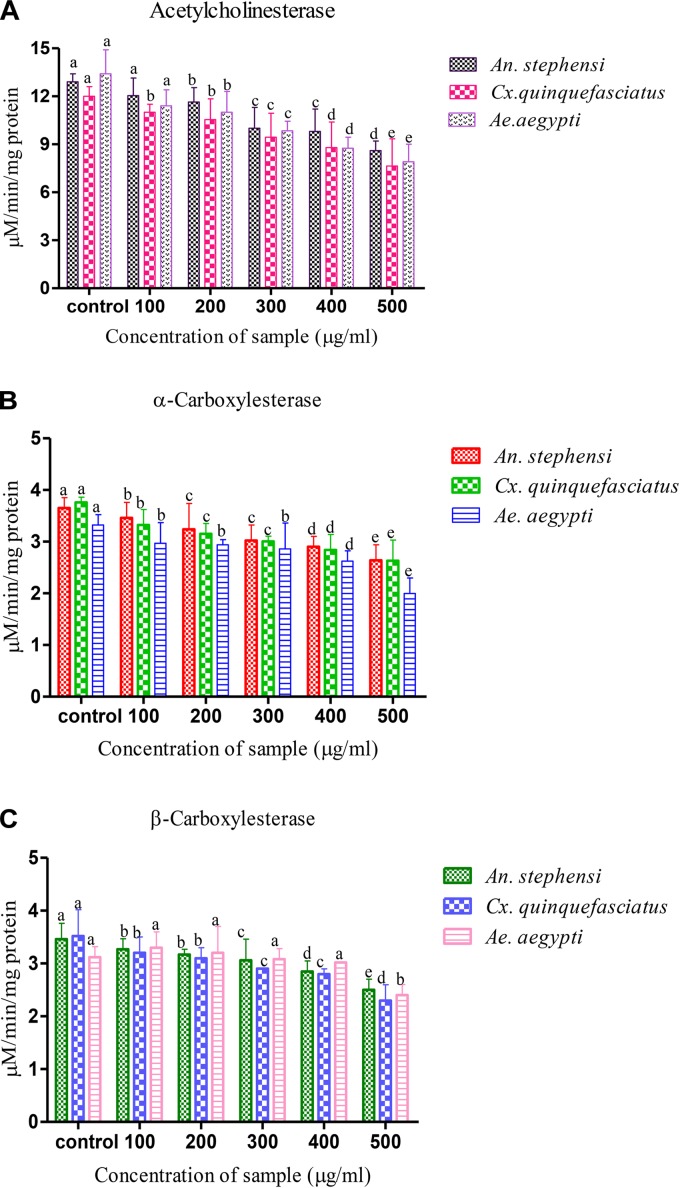
Biochemical analysis of **(A)** acetylcholine esterase, **(B,C)** α- and β- carboxylesterase *Aspergillus terreus* mycelium extract treated and untreated (Control) fourth instar larvae of vector mosquitoes *Anopheles stephensi, Culex quinquefasciatus* and *Aedes aegypti*. Statistical values followed by the same letter are not significantly differences according to Tukey test at *P* < 0.05 (one way ANOVA).

The biochemical constituent’s effects of *A. terreus* mycelium extract on the fourth instar larvae of tested mosquitoes were measured. The level of AchE activity was gradually decreased when the developing fourth instar larvae of tested mosquito at 24 h. The fungal metabolite exposure was significantly inhibited the larval AchE activity. The concentration of 100 μg/ml, *An. stephensi* larvae shown a maximum level of AchE expressed (*F* = 164.624, *P* < 0.05), followed by *Cx. quinquefasciatus* (*F* = 784.472, *P* < 0.05) and *Ae. aegypti* (*F* = 184.389, *P* < 0.05). Similarly, the higher concentration (500 μg/ml) of extract shown AchE activity was gradually declined in all tested larvae based on metabolite concentration. Over all, the obtained results revealed the enzyme expression is mainly dose dependent. The control larvae shown an enzyme level is normal. The larvae treated with *A. terreus* metabolite (at higher concentration), the AchE level was reported as twofold decreased against all tested mosquito (Figure 1A).

The decreased level of α-carboxylesterase activity of mosquitoes were noticed during the development of fourth instar larvae (Figures 1B,C), and it has reached the lowest level at 24 h. The exposure of larvae to the *A. terreus* mycelia metabolites had a dose-dependent activity on the level of β-carboxylesterase. The level of β-carboxylesterase activity also declined over the developmental period of fourth instar larvae of tested mosquitoes. Exposure of the larvae to metabolites significantly reduced α-carboxylesterase activity compared to control (3.651 to 2.641, 3.761 to 2.631, and 3.321 to 1.997 mg protein/ml of homogenate). A similar kind of observation was also made with β-carboxylesterase resulted decreased level of activity (3.461 to 2.531, 3.521 to 2.321, and 3.121 to 2.495 μM β-naphthol released/mg/min) against target mosquitoes.

**FIGURE 2:**
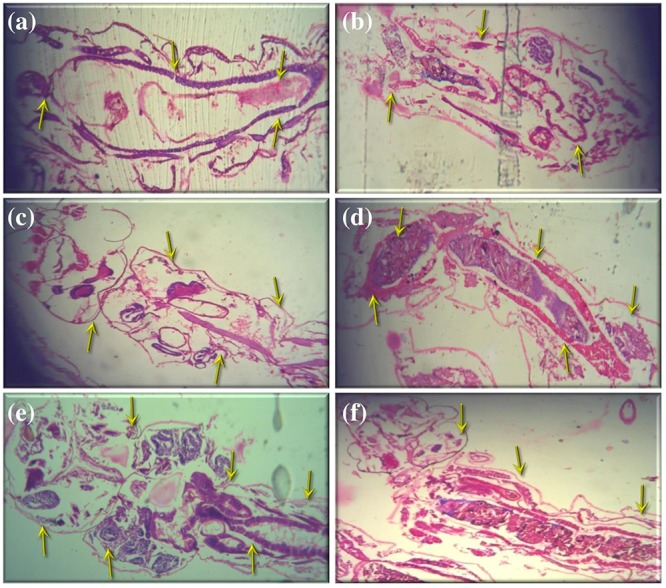
Histopathological analyses of treated and untreated fourth instar larvae of *An. stephensi, Cx. quinquefasciatus*, and *Ae. aegypti*: **(a,c**,**e)**. Control larvae **(b,d,f)**. *A. terreus* mycelium extract treated at (500 μg/ml) concentration against *An. stephensi, Cx. quinquefasciatus*, and *Ae. aegypti*. The yellow arrow indicates the whole abdominal region was collapse, mainly mid-gut and caeca, observed results revealed the loss of lateral and caudal hairs.

The histopathological analysis was performed to observe the maximum mortality rate from the fourth instar larvae of selected mosquitoes, during the 24 h exposure period. To the best of our knowledge, this is the first hand information on histopathological analysis of *A. terreus* mycelium ethyl acetate extracts against target mosquitoes. The control group showed the midgut epithelium consists of a single layer of digestive cells exhibiting well developed brush border and cytoplasm regions (Figures 2a,c,e). In this study, we observed the histological changes in the midgut, digestive tract, cortex, and epithelial cells of treated mosquito larvae.

The obtained results revealed that the highest concentration (500 μg/ml) of mycelia ethyl acetate extract from *A. terreus* shown disorganized epithelial layers and cells (Figure 2b) of *An. stephensi* larvae. In addition, *Cx. quinquefasciatus* treated with mycelium extract at higher concentration showed disintegration of abdomen region particularly the mid and hind gut and caeca, finally resulted the failure of lateral and caudal hairs (Figure 2d). The stereomicroscopic observations of fourth instar larvae of *Ae. aegypti* treated with *A. terreus* mycelium ethyl acetate extract noticed the loss of upper and lower head hair, antennal, and lateral hairs (Figure 2f). Furthermore, we observed the some parts of the mosquito larvae are severely affected, i.e., disintegration of epithelial layer and outer cuticle, highly damaged epithelium larvae, the vacuolated cells, the hindgut, and intracellular membranes.

The ovicidal activity results noticed zero percent of egg hatchability at specific concentrations of extract [for *An. stephensi* (500 μg/ml) (*F* = 12561.767; *P* < 0.05) and for *Cx. quinquefasciatus* (300 μg/ml) (*F* = 53079.779, *P* < 0.05) and *Ae. aegypti* (300 μg/ml) (*F* = 48493.715; *P* < 0.05)] (Table 2). The average egg hatchability percentage was observed, after 48 h treatment, and the result was directly linked with mycelium extract concentration. Whereas, the control showed 100% egg hatchability.

**Table 2:** Ovicidal activity of mycelia ethyl acetate extract of *A. terreus* against *An. stephensi* and *Cx. quinquefasciatus* and *Ae. aegypti*, after 48 h post experiment.

Mosquito species	Egg hatchability (%)^a^				
	
	Concentration (μg/ml)				
	
	Control	100	200	300	500
*An. Stephensi*	100.0 ± 0.0a	89.2 ± 1.5b	79.4 ± 1.5c	24.7 ± 0.2d	NH e
*Cx. quinquefasciatus*	100.0 ± 0.0a	92.1 ± 0.7b	76.7 ± 0.7c	NH d	NH d
*Ae. Aegypti*	100.0 ± 0.0a	88.1 ± 1.1b	74.2 ± 0.5c	NH d	NH d


*All value (X ± SD) depicts the means of the three values. NH-No hatchability (mortality 100%). ^*a*^Means in each row against each mosquito species followed by the difference letters are significantly different (*P* < 0.05, one-way ANOVA followed by Tukey’s test).*

**FIGURE 3:**
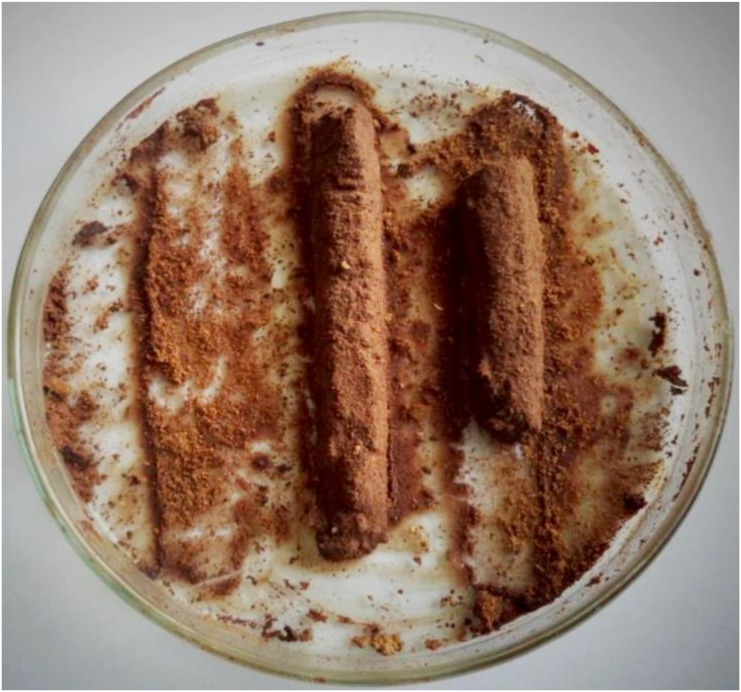
Preparation of mosquito coil from *A. terreus* mycelium ethyl acetate extract.

**FIGURE 4:**
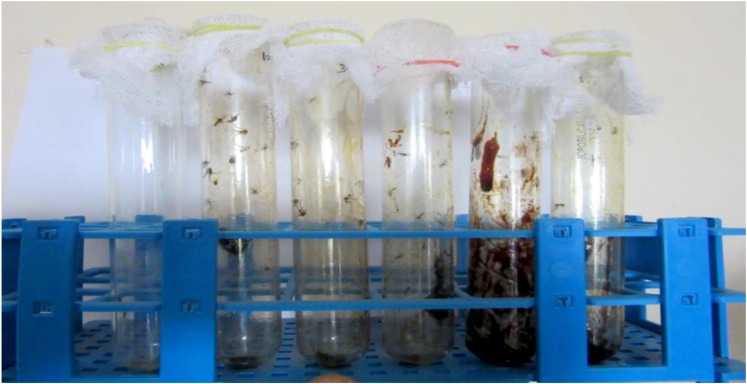
Adulticidal activity of ethyl acetate mycelia extract against *An. stephensi, Cx. quinquefasciatus*, and *Ae. aegypti* mosquitoes.

**FIGURE 5:**
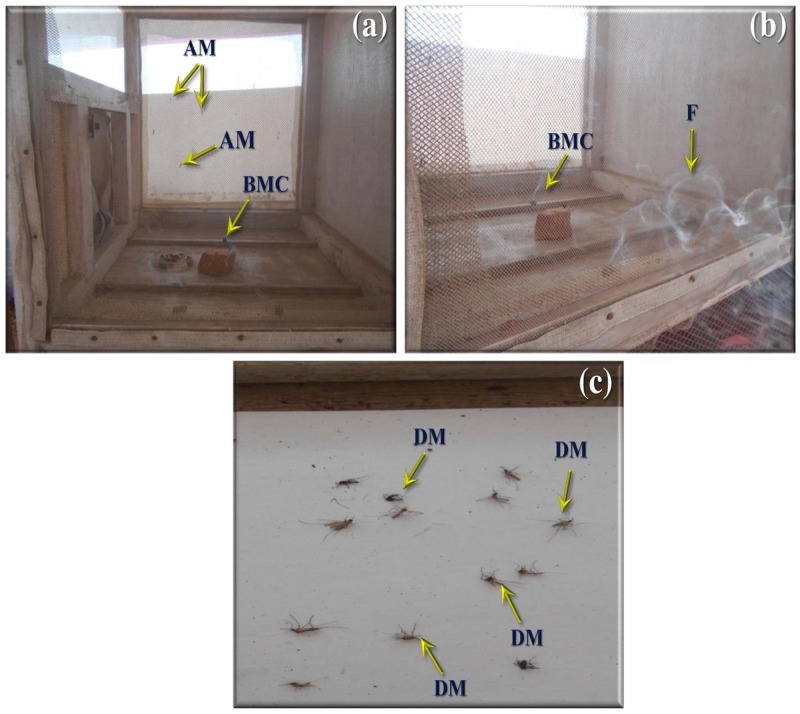
Smoke toxicity test for *A. terreus* ethyl acetate extract against *An. stephensi, Cx. quinquefasciatus*, and *Ae. aegypti*. **(a,b)** *A. terreus* mycelium ethyl acetate formulated mosquito coil. **(c)** Death mosquitoes after treatment of 40 min (AM, adult mosquitoes; BMC, burning mosquito coil; DM, dead mosquitoes).

Nil knocked down mosquitoes were observed during the exposure of the extract at 40 min (test and control groups) (Figure 3), due to the blank coil smoke and chamber condition (without coil) had no toxic effect against mosquitoes. The activity of mycelia ethyl acetate extract are depends on the complex mixture of active compounds. Adulticidal and smoke toxicity of ethyl acetate extract against malaria, filariasis, and dengue vectors and the results were presented in Figures 4, 5. The highest adulticidal activity was noticed on *An. stephensi, Cx. quinquefasciatus*, and *Ae. aegypti* with the LD_50_ values of 0.636 (0.001–5.376), 1.159 (0.002–7.977); 0.003 (0.000–0.823), 0.011 (0.000–1.370); and LD_90_ values of 0.336 (0.000–4.101), 0.785 (0.000–7.115), 0.232 (0.000–3.508), 0.563 (0.000–6.168) mg/ml, respectively. The control showed no mortality (Tables 3, 4). The higher concentration, the adult showed restless movement for several times, uncontrolled flying and finally died. The smoke toxicity was observed (after 40 min) and the mortality data were recorded after every 10 min time interval. The highest smoke toxicity was recorded in 40 min, than negative (without mycelia extract) and positive controls (commercial mosquito coil) of different mosquitoes, i.e., *Ae. aegypti* (91%) (*F* = 3570.425; *P* < 0.05), *Cx. quinquefasciatus* (89%) (*F* = 5585.69; *P* < 0.05) and *An. stephensi* (84%) (*F* = 4996.46; *P* < 0.05). The cent percent of mortality (100%) was obtained in the commercial mosquito control.

**Table 3:** Adulticidal activity of *A. terreus* mycelial extract against *An. stephensi, Cx. quinquefasciatus*, and *Ae. aegypti* mosquitoes.

Mosquitoes	Time intervals (min)	LD_50_ (LCL–UCL) 95% confidence limits (mg/ml)	LD_90_ (LCL–UCL) 95% confidence limits (mg/ml)	χ^2^ (*df* = 3)
*An. stephensi*	15 30	0.636 (0.001–5.376) 0.003 (0.000–0.823)	1.159 (0.002–7.977) 0.011 (0.000–1.370)	3.823 6.328
*Cx. quinquefasciatus*	15 30	0.336 (0.000–4.101) 0.785 (0.000–7.115)	0.232 (0.000–3.508) 0.563 (0.000–6.168)	7.134 7.223
*Ae. aegypti*	15 30	0.074 (0.000–4.802) 0.179 (0.000–4.802)	0.185 (0.000–5.800) 0.368 (0.000–7.117)	4.341 4.613


*LC_*50*_, lethal concentration 50% mortality; LC_*90*_, lethal concentration 90% mortality; LCL, lower confidence limits; UCL, upper confidence limits; df, degrees of freedom.*

**Table 4:** Smoke toxicity of *A. terreus* ethyl acetate extract on three major vectors.

Mosquito species	Observation after 40 min	^a^Mortality from *A. terreus* extract	Mortality from commercial mosquito coil (%)	Negative control (%)
*An. stephensi*	10	18.0 ± 1.1a	24.1 ± 0.3a	1
	20	47.1 ± 1.5b	50.0 ± 2.2b	1
	30	74.2 ± 1.1c	78.2 ± 1.3c	1
	40	84.2 ± 2.1d	94.3 ± 2.0d	0
*Cx. quinquefasciatus*	10	20.0 ± 0.9a	22.1 ± 2.3a	0
	20	48.1 ± 1.3b	52.2 ± 1.2b	1
	30	76.0 ± 2.1c	80.3 ± 2.3c	1
	40	89.6 ± 1.1d	100d	1
*Ae. aegypti*	10	21.0 ± 1.0a	25.1 ± 0.6a	1
	20	49.1 ± 0.9b	52.0 ± 2.0b	1
	30	77.3 ± 0.5c	83.2 ± 1.1c	0
	40	91.2 ± 1.7d	97.3 ± 1.0d	0


*^a^Means in each row against each mosquito species followed by the difference letters are significantly different (*P* < 0.05, one-way ANOVA followed by Tukey’s test).*

The effects of fungus mycelia extract have been proved as well smoke toxicity effect targeted mosquitoes. The mycelia ethyl acetate extracts exhibited a moderate toxic effect on the adult mosquitoes after 24 h of extract exposed. The results of this study indicate that *A. terreus* extract enhance in the smoke toxicity and it can be acted as effective alternative insecticide for *An. stephensi, Cx. quinquefasciatus*, and *Ae. aegypti* control. The smoke emitted from the extract exhibit a good knock down effect. However, when compared with the previous study (Suresh et al., 2015), the present investigation showed better smoke toxicity against the mosquito tested.

**FIGURE 6:**
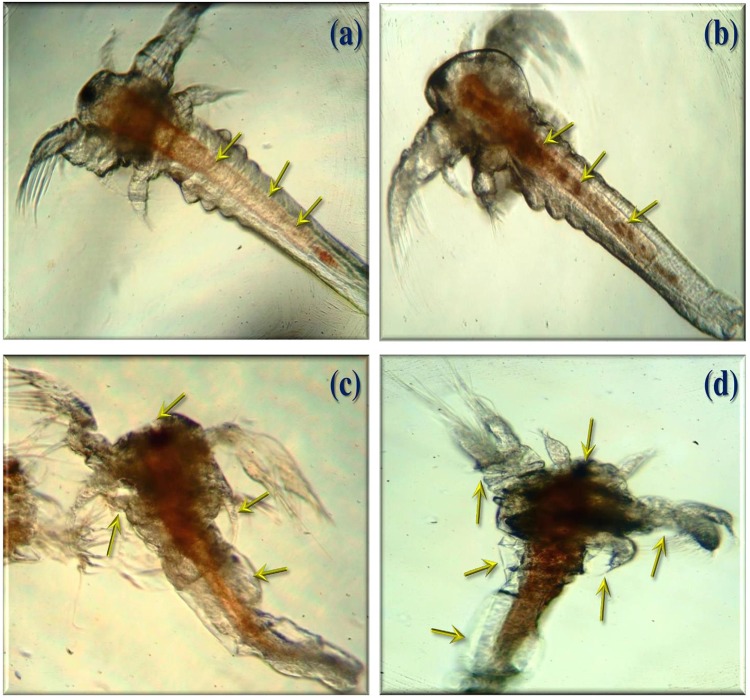
Ingestion of *A. terreus* mycelium ethyl acetate extracts to *Artemia nauplii* larvae (100 μg/ml) **(a)**. The empty guts in the control group of larvae **(b)**. Yellow arrow indicates the *A. nauplii* ingested with extract in the exposed larvae. **(c)** Morphological changes *A. nauplii* (eyeball shape, loss of eye color, and intestinal enlargement). **(d)** Loss of antennae and deformation of antennae.

**FIGURE 7:**
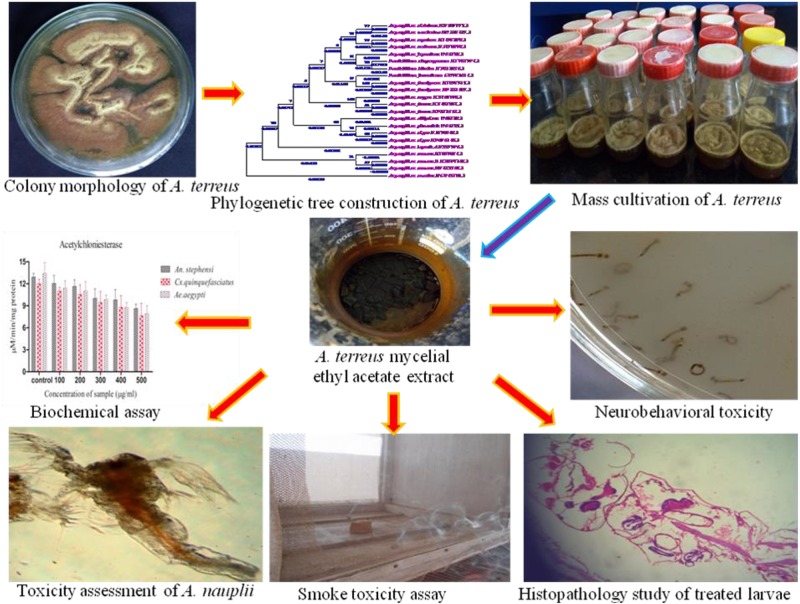
Schematic representation of overall study.

**FIGURE 8:**
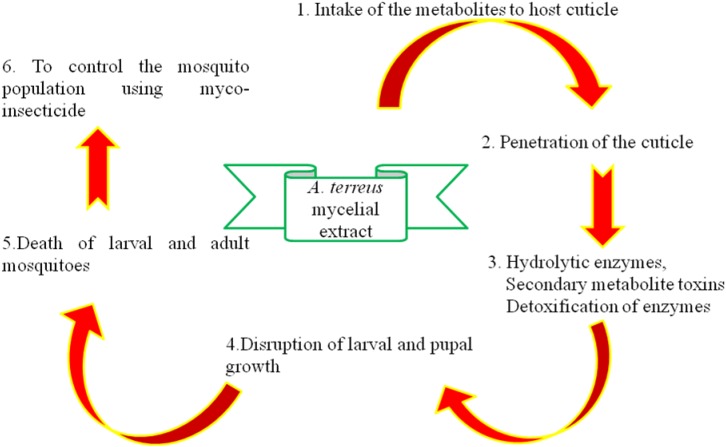
Possible mechanism pathway of mosquito control using *A. terreus.*

Biotoxicity assay of extract with *A. nauplii* of brine shrimp (suitable test aquatic organism) was carried out for the measurement of toxicity level. In control, *A. nauplii* showed an average speed of 2.96 mm s^-1^, after cultured in wells (8 and 48 h). After treated (at 24 h) with *A. terreus* extract, the swimming speed values were found to be 0.88 mm s^-1^. We observed behavioral changes in the swimming movement motionless phase and the *A. nauplii* position, after 8 h incubation treated with *A. terreus* mycelium extract. After 18 h exposure to the extract reflected slightly toxic cells, (either in exponential phase) and *A. nauplii* changes in swimming speed and their position on the water. The LC_50_ and LC_90_ values from the bioassay results of ethyl acetate extract against *A. terreus* strain was presented in Table 5. The better LC_50_ and LC_90_ values of *A. terreus* mycelia extract was noted against *A. nauplii* larvae treatments, i.e., 38.483, 27.595 (24 h) and 90.017, 53.710 μg/ml (48 h). The occurrence of dark yellow color substance in the gut region of the *Artemia* sp. treated with *A. terreus* mycelium ethyl acetate extract clearly indicates the accumulation of extract (Figure 6b). Whereas, *A. nauplii* exposed to seawater alone (as control) did not show any significant changes in the gut region (Figure 6a). We observed various changes in the *A. nauplii* during the exposure of extracts viz. changes in eye formation, eyeball shape and eyeball shrinking (Figure 6c), the intestinal swelling, malformations in the outer shell and antennae losses (Figure 6d). Schematic representation of overall study and mechanistic scheme were depicted in Figures 7, 8.

**Table 5:** Biotoxicity of *A. terreus* mycelia ethyl acetate extract on *Artemia nauplii.*

Concentrations (μg/ml)	12h			24h		
	LC_50_ (LCL–UCL) (μg/ml)	LC_90_ (LCL–UCL) (μg/ml)	χ^2^ (*df* = 13)	LC_50_ (LCL–UCL) (μg/ml)	LC_90_ (LCL–UCL) (μg/ml)	χ^2^ (*df* = 13)
100	38.483	90.017	1.251	27.595	53.710	1.481
200	(3.092–81.602)	(20.227–144.957)		(5.986–54.401)	(17.783–88.569)	
300						
400						
500						


*Control (salt H_*2*_O) – zero mortality. LC_*50*_, lethal concentration that kills 50% of the tested larvae; LC_*90*_, lethal concentration that kills 90% of the tested larvae; LCL, lower confidence limit; UCL, upper confidence limit; *df*, degree of freedom; χ^*2*^, chi-square values are significant at *P <* 0.05 level. Denote value of five replicates.*

## Discussion

DNA amplification and sequence analysis is a powerful tool in taxonomy studies (Rodrigues et al., 2007) of any living organisms. In present study, we used for the fungal universal primers for identification of ITS sequences in fungal rDNA and this process performed by PCR amplification method. After amplification, the PCR products were separated by using 1.2% agarose gel electrophoresis. The molecular size of sequences determined with the help of marker or control rDNA bands. The sequence result showed that the length of base pairs was found to be 555 bp. Recently, Ahmed et al. (2017) reported that the morphological and molecular level identification of *Aspergillus* sp. using the universal primer ITS1 and ITS4 region with amplification size of 600 bp. Similarly, Gehlot et al. (2011) stated that the identification of *Aspergillus* sp. using ITS1-5.8S rDNA-ITS2 sequences. Likewise, Henry et al. (2000) reported the amplification fragments ranged from 565 to 613 bp for various *Aspergillus* sp, which was shown 595 bp in *A. flavus*.

Mycoinsecticides are better as well as alternative insecticidal agents due to their least contamination level, low toxicity to humans and various other advantages (Liu et al., 2000). Abamectins, milbemectin, and spinosyns were developed as bio-insecticides (Seiber et al., 2014) from the microbial source. Bioassay screening of *A. terreus* mycelium ethyl acetate extract exhibited various bioactivities against the fourth instar larvae of *An. stephensi, Cx. quinquefasciatus*, and *Ae. aegypti*. Rapid and constant behavioral toxicity symptoms were observed during the course of investigation, i.e., tremors and muscle paralysis, considerable increase in larvae knock-down rate, leads to a strong neurotoxic action. These symptoms consist of hyper-activity, convulsions, and tremors followed by paralysis (knock-down), similar to caused by chemical insecticides (organophosphate or carbamate). Interestingly, Muema et al. (2016) observed the *Ageratum conyzoides* extract remarkably accelerated the growth of *Anopheles* sp. larvae into pupae resulted into deficient melanization and uneven dead larval-pupal intermediates. Similarly, Sharma et al. (2015) reported the *Achyranthes aspera* extract treated with *Ae. aegypti* larvae showed modified behavioral symptoms viz: excitation and restlessness, observe violent anal self-biting behavior. Likewise, Choochote et al. (2004) observed an abnormal and irregular movement of *Ae. aegypti* larvae treated with the *Apium graveolens* extract.

We observed the larvae having symptoms of tremors and muscle paralysis are the major sign related to neurobehavioral toxicity. The excessive wriggling movements made by *Ae. aegypti* larvae while treating with metabolite than untreated larvae (Baatrup and Bayley, 1993). *A. terreus* mycelium ethyl acetate extract showed promising larval bioactivity for the control of *Ae. aegypti* larvae in terms of behavioral, larvicidal toxicity, disrupting the growth, molting, and morphological changes. The anal gills of tested larvae served as major site for Na^+^, Cl^-^, and K^+^ uptake, enhance the role of the Malpighian tubules and rectum. The *A. terreus* mycelia metabolites may interrupt the ion transport due to damage in the anal papillae and an outer cuticle layer of tested larvae (Perumalsamy et al., 2013). In our study, we observed 39% dead fourth instar larvae (at 100 μg/ml) and 4% of pupae death (200 and 300 μg/ml) treated with mycelium extract, showed maximum level of morphological and growth/molting associated deformities. The morphological deformities of fourth instar larvae shown like a black and pigmented cuticles in the thoracic and abdomen region of pupae resulted abnormal cuticle melanization, due to inhibition of chitin synthesis. To the best of our knowledge, there is no previous report available on the neurobehavioral toxicity and knock down effects of fungal derived extract or metabolites against the mosquito larvae. Similar observations were made by Bream et al. (2010) and Soonwera and Phasomkusolsil (2016) with abnormalities of *Cx. pipiens, Ae. aegypti*, and *An. dirus* larval morphology such as pigmented, deformed larvae, incomplete eclosion, white pupae, deformed pupae, dead normal pupae and incomplete pupal eclosion after exposure to some plant oils. The outcome of results was supported by previous findings of Saxena et al. (1994) reported that the morphological deformities of mosquitoes (including darkening of the larval cuticle, during molting and development of *Cx*. *quinquefasciatus*) induced by *Ageratum conyzoides* extract.

The fourth instar larvae of selected mosquitoes (*An. stephensi, Cx. quinquefasciatus*, and *Ae. aegypti*) treated with mycelium extract obtained from *A. terreus* (500 μg/ml) showed morphological and behavioral changes. After 30 min of exposure, the larvae were found be restless and exhibited sluggish movements, while increasing exposure time. The treated larvae showed morphological changes in the anal papillae region and cuticle layer. Larvae treated with *A. terreus* mycelium ethyl acetate extract developed dramatic lesions affected mainly in the epithelial layer of the midgut than the control. The cross section of the larvae midgut illustrates disruption in the appearance of columnar cells, swelling, and extruding masses of cellular molecules in the mid gut position. Recently, Abinaya et al. (2018) examined the *Bacillus licheniformis* exopolysaccharide treated *An. stephensi* and *Ae. aegypti* larval showed histological damages of midgut, muscles, and shrinkage in the abdominal regions. Likewise, Seetharaman et al. (2017) reported the limonoid compound from *Penicillium oxalicum* showed the damage of microvilli, midgut lumen, peritrophic membrane, and epithelial cells of tested *Culex* sp. mosquito larvae. Interestingly, Bawin et al. (2016) reported that the histopathological effects of *Aspergillus clavatus* metabolites against *Cx. quinquefasciatus* larvae. Similarly, Elumalai et al. (2016) studied the histopathology alteration of isolated catechin larvicidal compound against fourth instar larvae of *Ae. aegypti, An. Stephensi*, and *Cx. quinquefasciatus.* Recently, Ragavendran et al. (2017) reported that the midgut cells of *Ae. aegypti* (fourth-instar larvae) had swelling in the gut lumen, reducing intercellular contacts and degeneration of nuclei, after treated with *P. daleae* mycelium extract.

Different types of enzymes namely oxidases, reductases, and esterases used by the mosquito for detoxifying the pesticide compounds (Senthil-Nathan et al., 2007, 2008; Waliwitiya et al., 2012). Among them, acetylcholine esterase enzyme breaks down the neurotransmitter acetylcholine at the synaptic cleft. The nerve impulse can be transported across the gap. Neurotransmitters must be cleaned immediately after the message is passed, and if not, the larva causes paralysis (Fouad et al., 2017). Esterases are the key enzymes that are responsible for resistance mechanism against chemical/synthetic insecticides in mosquitoes (Brogdon and McAllister, 1998) by cleaving the carboxyl ester and phosphodiester bonds. Resistant insects generally demonstrate a very high activity of esterases (Lemos et al., 1996; Ranson and Hemingway, 2005). Similarly, Koodalingam et al. (2012) reported a significant decrease in acetylcholinesterase levels in *Ae. aegypti* larvae treated with the Bt-based product (Vectobar). The present study results clearly stated that a significant reduction of acetylcholinesterase level activity in the larvae, treated with *A. terreus* mycelium ethyl acetate extract than control larvae. Similarly, Fan et al. (2013) reported that enzymatic levels of esterase (EST) and glutathione S-transferase (GST) were notably increased when *Dendrolimus tabulaeformis* larvae treated with *Beauveria bassiana* spore suspension.

The mosquito (*An. stephensi, Cx. quinquefasciatus*, and *Ae. aegypti*) larval homogenates were exposed to *A. terreus* mycelia extract found to be the disturbed physiological constituents are evident for reduction of detoxification enzymes. This observation suggests that direct toxic effect of *A. terreus* mycelia ethyl acetate metabolites in the protein synthetic machinery of the mosquito larvae. The α- and β-carboxylesterase activity level was gradually decreased while the formation of fourth instar larvae of three mosquitoes. The exposure of larvae to the *A. terreus* mycelia metabolite had a dose dependent manner at the level of both carboxylesterase activities. Likewise, Koodalingam et al. (2012) observed dose-dependent level of α- and β-carboxylesterase activity and considerable alteration in the isoenzyme of *Ae. aegypti* larvae by Laranja et al. (2003).

The zero percentage of ovicidal and egg hatchability results were observed in all tested mosquitoes, i.e., *An. stephensi* (500 μg/ml), *Cx. quinquefasciatus* (300 μg/ml), and *Ae. aegypti* (300 μg/ml). The average percent of hatchability of eggs were observed, after 48 h treatment. Overall, the ovicidal activity was decreased with an increase in concentration of *A. terreus* mycelium ethyl acetate extract. Also, Luz et al. (2007) investigated the ovicidal effects of hyphomycete fungi (21 Nos) against the eggs of *Ae. aegypti*. Similarly, Karthik et al. (2011) reported that the actinobacteria (LK-2 and LK-3) derived crude extract against *Cx. gelidus* and *Cx. tritaeniorhynchus* revealed 100% ovicidal (no egg hatchability) observed in 1,000 ppm.

In contrast, *A. terreus* mycelial extract was showed good adulticidal activity against *Ae. aegypti* the maximum LD_50_ and LD_90_ values found than the *Cx. quinquefasciatus* and *An. stephensi* adults. Mohanty et al. (2008) proved the efficiency of *F. pallidoroseum* spores against adult mosquitoes. Whereas, the present investigation studied *A. terreus* mycelia ethyl acetate metabolite having remarkable adulticidal effects against three targeted mosquitoes. The maximum smoke toxicity was recorded (within 40 min) of different mosquitoes, i.e., *Ae. aegypti* (91%), *Cx. quinquefasciatus* (89%), and *An. stephensi* (84%), while commercial mosquito coil showed 100% adult mortality. Recently, Balasubramani et al. (2018) reported the smoke toxicity assay of *Salmonella bongori* metabolites showed 62% mortality against *Cx. quinquefasciatus* compared with positive control (pyrethrin). Also, Dua et al. (2010) identified the essential oil from plants having adult knockdown properties against targeted mosquitoes.

Biotoxicity assay of fungal metabolites was conducted with brine shrimp *A. nauplii*. Meyer et al. (1982) and Nguta et al. (2012) categorized the biotoxicity assay results based on dose/concentration of extracts, i.e., non-toxic (>1000 μg/ ml), weakly toxic (500–1000 μg/ml), moderate toxic (100–500 μg/ml) and strong toxic (<100 μg/ml) against tested insects. In control, *A. nauplii* show a normal swimming speed in the wells. After 24 h treated with mycelia ethyl extract of *A. terreus*, the swimming speed of organisms has changed as motionless. Likewise, Charoy et al. (1995), Charoy and Janssen (1999), and Larsen et al. (2008) investigated the treatment effect of toxic metabolites on *Artemia* sp. Diana et al. (2011) studied the *Fusarium* isolates (No: 24) exhibit various levels of toxicity on the brine shrimp, based on the role of fusaproliferin and beauvericin compounds (Bosch et al., 1989; Altomare et al., 1995; Moretti et al., 2007). The present investigation reveals that the uptake with *A. terreus* mycelium ethyl acetate extract treated with *A. nauplii* shown an accumulation of toxin in the mid-gut region leads to the brine shrimp death. Recently, Ragavendran et al. (2017) identified *Penicillium daleae* mycelia metabolites and tested for *A. nauplii* alterations of body structures and loss of antenna with the LC_50_ and LC_90_ value 0.290 and 0.609 μg/ml, respectively. Similarly, Ruhul Amin et al. (2003) reported the LC_50_ value (17.78 μg/ml) of *Penicillium* sp. extract against the brine shrimp mortality, which is quite lower toxicity. Likewise, Miao et al. (2012) found that the LC_50_ value of 6-O-methylaverufin (produced by the endophytic fungus *A. versicolor*) was 0.5 μg/ml. Another study, An et al. (2013) reported that 4-phenyl-3,4-dihydroquinolone and aflaquinolone derivatives produced by *A. nidulans* exhibited strong toxicity against *A. salina*, with LC_50_ values ranged from 4.5 to 7.1 μM.

## Conclusion

A fungal strain isolated from soil was identified as *A. terreus* (KX694148.1) through ITS rDNA sequencing and phylogenetic tree analysis. The present study revealed that significant to moderate toxic effect of *A. terreus* mycelia extract on different stages of mosquitoes such as egg, larvae, pupa, and adults of *Ae. aegypti, Cx. quinquefasciatus*, and *An. stephensi*. *A. terreus* extract exhibited strong neurobehavioral toxicity, knock-down effect, the ovicidal and adulticidal effect on *An. stephensi, Cx. quinquefasciatus*, and *Ae. aegypti* in a dose and time-dependent manner. The exposure of *A. terreus* extract caused significant behavioral changes, growth disturbance and morphological abnormalities on tested mosquito species. The activities of vital mosquito enzymes such as α- and β-carboxylesterase and AchE were decreased upon *A. terreus* extract treatment. The histopathological study shows *A. terreus* extract affected the larval development and completely damaged the digestive system of mosquito. The smoke of *A. terreus* extract induced the time-dependent adult death/toxicity and behavioral changes in mosquitoes. *A. terreus* extract showed considerable toxic effect, behavioral changes and anomalies on *A. nauplii* larvae. The outcome of the present investigation concludes that ethyl acetate extract of *A. terreus* contains promising mosquito control principles which may be used in the control of mosquito in the future. Further studies on isolation and identification of bioactive constituents from *A. terreus* extract is under process.

## Ethics Statement

All applicable national, international, and/or institutional standard protocols for the concern and utilized the insects/crustaceans were maintained and experiments conducted.

## Author Contributions

CR and DN planned the research, performed the experiments, shared chemicals and analysis tools, and wrote the manuscript. CR analyzed the statistics. RS and MK contributed significant work pertaining to the analysis of data and finally, all the authors has read and approved the final manuscript.

## Conflict of Interest Statement

The authors declare that the research was conducted in the absence of any commercial or financial relationships that could be construed as a potential conflict of interest.

## References

[B1] Abbott W. S. (1925). A method of computing the effectiveness of an insecticide. *J. Econ. Entomol.* 18 265–267. 10.1093/jee/18.2.265a

[B2] Abinaya M. Vaseeharan B. Divya M. Vijayakumar S. Govindarajan M. Alharbi N. S. (2018). Structural characterization of *Bacillus licheniformis* Dahb1 exopolysaccharide—antimicrobial potential and larvicidal activity on malaria and Zika virus mosquito vectors. *Environ. Sci. Pollut. Res.* 25 18604–18619. 10.1007/s11356-018-2002-6 29704178

[B3] Abreu J. Gonzalez J. Jaqueman F. (2003). Conservación por liofilación de diferentes especies de géneros de levaduras. *Alimentaria* 40 119–122.

[B4] Ahmed D. A. Al-Khafaji N. J. Ahmed L. T. (2017). Isolation and molecular identification of *Aspergillus* spp. collected from different sources of animals feed. *Int. J. Curr. Microbiol. App. Sci.* 6 1792–1797. 10.20546/ijcmas.2017.606.208

[B5] Altomare C. Logrieco A. Bottalico A. Mulé G. Moretti A. Evidente A. (1995). Production of type A trichothecenes and enniatin B by *Fusarium sambucinum* Fuckel sensu lato. *Mycopathologia* 129 177–181. 10.1007/BF01103344 7566055

[B6] Alyuruk H. Demir G. K. Cavas L. (2013). A video tracking based improvement of acute toxicity test on *Artemia salina*. *Mar. Freshw. Behav. Physiol.* 46 251–266. 10.1080/10236244.2013.814224

[B7] An C. Y. Li X. M. Luo H. Li C. S. Wang M. H. Xu G. M. (2013). 4-Phenyl-3, 4-dihydroquinolone derivatives from *Aspergillus nidulans* MA-143, an endophytic fungus isolated from the mangrove plant *Rhizophora stylosa*. *J. Natur. Prod.* 76 1896–1901. 10.1021/np4004646 24099304

[B8] Atalla M. M. Elkhrisy E. A. M. Asem M. A. (2011). Production of textile reddish brown dyes by fungi. *Malays J. Microbiol.* 7 33–40.

[B9] Ates M. Daniels J. Arslan Z. Farah I. O. (2013). Effects of aqueous suspensions of titanium dioxide nanoparticles on *Artemia salina*: assessment of nanoparticle aggregation, accumulation, and toxicity. *Environ. Monit. Asses.* 185 3339–3348. 10.1007/s10661-012-2794-7 22810381 PMC3491177

[B10] Baatrup E. Bayley M. (1993). Quantitative analysis of spider locomotion employing computer-automated video tracking. *Physiol. Behav.* 54 83–90. 10.1016/0031-9384(93)90047-J 8327613

[B11] Balasubramani G. Deepak P. Aiswarya D. Karthik Raja R. Kamaraj C. Perumal P. (2018). Multipurpose efficacy of the lyophilized cell-free supernatant of *Salmonella bongori* isolated from the freshwater fish, *Devario aequipinnatus*: toxicity against microbial pathogens and mosquito vectors. *Environ. Sci. Pollut. Res.* 25 29162–29180. 10.1007/s11356-018-2838-9 30112646

[B12] Balaraman K. Bheema Rao U. S. Rajagopalan P. K. (1979). Isolation of *Metarrhizium anisopliae, Beauveria tenella* and *Fusarium oxysporum* (Deuteromycetes) and their pathogenicity to *Culex fatigans* and *Anopheles stephensi*. *Ind. J. Med. Res.* 70 718–722. 535971

[B13] Bawin T. Seye F. Boukraa S. Zimmer J. Y. Raharimalala F. N. Ndiaye M. (2016). Histopathological effects of *Aspergillus clavatus* (Ascomycota: Trichocomaceae) on larvae of the southern house mosquito, *Culex quinquefasciatus* (Diptera: Culicidae). *Fungal Boil.* 120 489–499. 10.1016/j.funbio.2016.01.002 27020151

[B14] Benelli G. Mehlhorn H. (2016). Declining malaria, rising dengue and Zika virus: insights for mosquito vector control. *Parasitol. Res.* 115 1747–1754. 10.1007/s00436-016-4971-z 26932263

[B15] Benelli G. Rajeswary M. Govindarajan M. (2018). Towards green oviposition deterrents? Effectiveness of *Syzygium lanceolatum* (Myrtaceae) essential oil against six mosquito vectors and impact on four aquatic biological control agents. *Environ. Sci. Pollut. Res.* 25 10218–10227. 10.1007/s11356-016-8146-3 27921244

[B16] Benelli G. Romano D. (2017). Mosquito vectors of Zika virus. *Entomol. General.* 36 309–318. 10.1127/entomologia/2017/0496

[B17] Bigelis R. Arora D. K. (1992). “Organic acids of fungi,” in *Handbook of Applied Mycology: Fungal biotechnology* Vol. 4 eds Arora D. K. Elander R. P. Murekji K. G. (New York, NY: Marcel Dekker Inc), 357–376.

[B18] Boruta T. Bizukojc M. (2016). Induction of secondary metabolism of *Aspergillus terreus* ATCC 20542 in the batch bioreactor cultures. *Appl. Microbiol. Biotechnol.* 100 3009–3022. 10.1007/s00253-015-7157-1 26603760 PMC4786612

[B19] Bosch U. Mirocha C. J. Abbas H. K. Di Menna M. (1989). Toxicity and toxin production by *Fusarium* isolates from New Zealand. *Mycopathologia* 108 73–79. 10.1007/BF00436056 2594049

[B20] Bream A. S. El-Sheikh T. M. Y. Fouda M. A. Hassan M. I. (2010). Larvicidal and repellent activity of extracts derived from aquatic plant *Echinochloa stagninum* against *Culex pipiens*. *Tunis J. Plant Prot.* 5 107–124.

[B21] Brogdon W. G. McAllister J. C. (1998). Insecticide resistance and vector control. *Emerg. Infectious Dis.* 4:605. 10.3201/eid0404.980410 9866736 PMC2640263

[B22] Caraballo H. King K. (2014). Emergency department management of mosquito-borne illness: malaria, dengue, and West Nile virus. *Emerg. Med. Pract.* 16 1–23. 25207355

[B23] Casanova H. Ortiz C. Peláez C. Vallejo A. Moreno M. E. Acevedo M. (2002). Insecticide formulations based on nicotine oleate stabilized by sodium caseinate. *J. Agric. Food Chem.* 50 6389–6394. 10.1021/jf0257244 12381122

[B24] Charoy C. Janssen C. R. (1999). The swimming behaviour of *Brachionus calyciflorus* (rotifer) under toxic stress. II. Comparative sensitivity of various behavioural criteria. *Chemosphere* 38 3247–3260. 10.1016/j.chemosphere.2013.08.086 24079998

[B25] Charoy C. P. Janssen C. R. Persoone G. Clement P. (1995). The swimming behaviour of *Brachionus calyciflorus* (rotifer) under toxic stress. I. The use of automated trajectory for determining sub-lethal effects of chemicals. *Aquat. Toxicol.* 32 271–282. 10.1016/j.chemosphere.2013.08.086 24079998

[B26] Cheng S. S. Huang C. G. Chen W. J. Kuo Y. H. Chang S. T. (2008). Larvicidal activity of tectoquinone isolated from red heartwood-type *Cryptomeria japonica* against two mosquito species. *Biores. Technol.* 99 3617–3622. 10.1016/j.biortech.2007.07.038 17804221

[B27] Choochote W. Tuetun B. Kanjanapothi D. Rattanachanpichai E. Chaithong U. Chaiwong P. (2004). Potential of crude seed extract of celery, *Apium graveolens L.*, against the mosquito *Aedes aegypti (L.) (Diptera: Culicidae).* *J. Vector Ecol.* 29 340–346. 15707293

[B28] Colovic M. B. Krstic D. Z. Lazarevic-Pasti T. D. Bondzic A. M. Vasic V. M. (2013). Acetylcholinesterase inhibitors: pharmacology and toxicology. *Cur. Neuropharmacol.* 11 315–335. 10.2174/1570159x11311030006 24179466 PMC3648782

[B29] Dev V. Sharma V. P. Barman K. (2015). Mosquito-borne diseases in assam, north-east India: current status and key challenges. *WHO South-East Asia J. Public Health* 4 20–29. 10.4103/2224-3151.206616 28607271

[B30] Diana C. Tan Flematti G. R. Ghisalberti E. L. Sivasithamparam K. Chakraborty S. Obanor F. (2011). Mycotoxins produced by *Fusarium* species associated with annual legume pastures and ‘sheep feed refusal disorders’ in Western Australia. *Mycotoxin Res.* 27 123–135. 10.1007/s12550-010-0085-0 23605703

[B31] Dikmen M. Canturk Z. Engur S. Kaya Tilki E. (2017). Inhibitory effects of secondary metabolites of halotolerant *Aspergillus terreus* on angiogenesis. *Biomed. Res.* 28 3613–3618.

[B32] Dua V. K. Pandey A. C. Dash A. P. (2010). Adulticidal activity of essential oil of *Lantana camara* leaves against mosquitoes. *Ind. J. Med. Res.* 131 434–439. 20418559

[B33] Ellman G. L. Courtney K. D. Andres V. Jr. Featherstone R. M. (1961). A new and rapid colorimetric determination of acetylcholinesterase activity. *Biochem. Pharmacol.* 7 88–95. 10.1016/0006-2952(61)90145-9 13726518

[B34] Elumalai D. Hemavathi M. Hemalatha P. Deepaa C. V. Kaleena P. K. (2016). Larvicidal activity of catechin isolated from *Leucas aspera* against *Aedes aegypti, Anopheles stephensi*, and *Culex quinquefasciatus* (Diptera: Culicidae). *Parasitol. Res.* 115 1203–1212. 10.1007/s00436-015-4856-6 26711450

[B35] EPA (2002). *Methods for Measuring the Acute Toxicity of Effluents and Receiving Waters to Freshwater and Marine Organisms*, 5th Edn. Available at: http://water.epa.gov/scitech/methods/cwa/wet/disk2_index.cfm

[B36] Faimali M. Garaventa F. Piazza V. Greco G. Corra C. Magillo F. (2006). Swimming speed alteration of larvae of *Balanus amphitrite* as a behavioural end-point for laboratory toxicological bioassays. *Mar. Biol.* 149 87–96. 10.1007/s00227-005-0209-9

[B37] Fan J. Xie Y. Xue J. Liu R. (2013). The effect of *Beauveria brongniartii* and its secondary me-tabolites on the detoxification enzymes of the pine caterpillar, *Dendrolimus tabulaeformis*. *J. Insect Sci.* 13:44. 10.1673/031.013.4401 23909949 PMC3740923

[B38] Finney D. J. (1971). *Probit Analysis.* London: Cambridge University Press, 68–78.

[B39] Fouad H. Hongjie L. Hosni D. Wei J. Abbas G. Ga’al H. (2017). Controlling *Aedes albopictus* and *Culex pipiens* pallens using silver nanoparticles synthesized from aqueous extract of Cassia fistula fruit pulp and its mode of action. *Artificial Cells Nanomed. Biotechol.* 46 558–567. 10.1080/21691401.2017.1329739 28541740

[B40] Gao H. Guo W. Wang Q. Zhang L. Zhu M. Zhu T. (2013). Aspulvinones from a mangrove rhizosphere soil-derived fungus *Aspergillus terreus* Gwq-48 with anti-influenza A viral (H1N1) activity. *Bioorg. Med. Chem. Lett.* 23 1776–1778. 10.1016/j.bmcl.2013.01.051 23411074

[B41] Garaventa F. Gambardella C. Di Fino A. Pittore M. Faimali M. (2010). Swimming speed alteration of *Artemia* sp. and *Brachionus plicatilis* as a sub-lethal behavioural end-point for ecotoxicological surveys. *Ecotoxicoloy* 19 512–519. 10.1007/s10646-010-0461-8 20099027

[B42] Gehlot P. Purohit D. K. Singh S. K. (2011). Molecular diagnostic of human pathogenic *Aspergillus* species. *Ind. J. Biotechnol.* 10 207–211.

[B43] Gopalakrishnan R. Baruah I. Vee V. (2014). Monitoring of malaria, Japanese encephalitis and filariasis vectors monitoring of malaria, Japanese encephalitis and filariasis vectors. *Med. J. Armed Forces Ind.* 70 129–133. 10.1016/j.mjafi.2013.10.014 24843200 PMC4017167

[B44] Guo C. J. Knox B. P. Chiang Y. M. Lo H. C. Sanchez J. F. Lee K. H. (2012). Molecular genetic characterization of a cluster in *A. terreus* for biosynthesis of the meroterpenoid terretonin. *Organic Lett.* 14 5684–5687. 10.1021/ol302682z 23116177 PMC3538129

[B45] Guo C. J. Wang C. C. (2014). Recent advances in genome mining of secondary metabolites in *Aspergillus terreus*. *Front. Microbiol.* 5:717. 10.3389/fmicb.2014.00717 25566227 PMC4274970

[B46] Hawkes N. J. Janes R. W. Hemingway J. Vontas J. (2005). Detection of resistance-associated point mutations of organophosphate-insensitive acetylcholinesterase in the olive fruit fly, *Bactrocera oleae* (Gmelin). *Pest. Biochem Physiol.* 81 154–63. 10.1016/j.pestbp.2004.11.003 12144698

[B47] Hemingway J. Karunaratne H. P. P. (1998). Mosquito carboxylesterases: a review of the molecular biology and biochemistry of a major insecticide resistance mechanism. *Med. Vet. Entomol.* 12 1–12. 10.1046/j.1365-2915.1998.00082.x 9513933

[B48] Henry T. Iwen P. C. Hinrichs S. H. (2000). Identification of *Aspergillus* species using internal transcribed spacer regions 1 and 2. *J. Clin. Microbiol.* 38 1510–1515. 10747135 10.1128/jcm.38.4.1510-1515.2000 PMC86477

[B49] Iheanacho H. E. Njobeh P. B. Dutton F. M. Steenkamp P. A. Steenkamp L. Mthombeni J. Q. (2014). Morphological and molecular identification of filamentous *Aspergillus flavus* and *Aspergillus parasiticus* isolated from compound feeds in South Africa. *Food. Microbiol.* 44 180–184. 10.1016/j.fm.2014.05.019 25084661

[B50] Ikezawa H. Taguchi R. (1981). “Phosphatidylinositol-specific phospholipase C from *Bacillus cereus* and *Bacillus thurinǵiensis*,” in *Methods in Enzymology*, Vol. 71 eds Colowick S. P. Kaplan N. O. (Cambridge, MA: Academic Press), 731–741.

[B51] Karthik L. Gaurav K. Rao K. B. Rajakumar G. Rahuman A. A. (2011). Larvicidal, repellent, and ovicidal activity of marine actinobacteria extracts against *Culex tritaeniorhynchus* and *Culex gelidus*. *Parasitol. Res.* 108 1447–1455. 10.1007/s00436-010-2193-3 21153420

[B52] Khattak S. U. Iqbal Z. Lutfullah G. Bacha N. Khan A. A. Saeed M. (2014). Phytotoxic and herbicidal activities of *Aspergillus* and *Penicillium* species isolated from rhizosphere and soil. *Pak. J. Weed Sci. Res.* 20 293–303.

[B53] Klich M. A. Pitt J. I. (1992). *A Laboratory Guide Common Aspergillus Species and Their Teleomorphs.* Canberra, ACT: Commonwealth Scientific and Industrial Research Organisation, Division of Food Processing.

[B54] Kokkali V. Katramados I. Newman J. D. (2011). Monitoring the effect of metal ions on the mobility of *Artemia salina* nauplii. *Biosensors* 1 36–45. 10.3390/bios1020036 25586826 PMC4264340

[B55] Koodalingam A. Mullainadhan P. Rajalakshmi A. Deepalakshmi R. Ammu M. (2012). Effect of a Bt-based product (Vectobar) on esterases and phosphatases from larvae of the mosquito *Aedes aegypti*. *Pest. Biochem. Physiol.* 104 267–272. 10.1016/j.pestbp.2012.09.008

[B56] Kumar S. Tamura K. Nei M. (2004). MEGA3: integrated software for molecular evolutionary genetics analysis and sequence alignment. *Brief. Bioinformat.* 5 150–163. 10.1093/bib/5.2.150 15260895

[B57] Lacey C. M. Lacey L. A. Roberts D. R. (1988). Route of invasion and histopathology of *Metarhizium anisopliae* in *Culex quinquefasciatus*. *J. Invert Pathol.* 52 108–118. 10.1016/0022-2011(88)90109-7 3418133

[B58] Laranja A. T. Manzatto A. J. Campos Bicudo H. E. M. D. (2003). Effects of caffeine and used coffee grounds on biological features of *Aedes aegypti* (Diptera, Culicidae) and their possible use in alternative control. *Genet. Mol. Biol.* 26 419–429. 10.1590/S1415-47572003000400004

[B59] Larsen P. S. Madsen C. V. Riisgård H. U. (2008). Effect of temperature and viscosity on swimming velocity of the copepod *Acartia tonsa*, brine shrimp *Artemia salina* and rotifer *Brachionus plicatilis*. *Aquat. Biol.* 4 47–54. 10.3354/ab00093

[B60] Lemos F. J. Cornel A. J. Jacobs-Lorena M. (1996). Trypsin and aminopeptidase gene expression is affected by age and food composition in *Anopheles gambiae*. *Insect. Biochem. Mol. Biol.* 26 651–658. 10.1016/S0965-1748(96)00014-8 8995788

[B61] Liu S. Q. Shi J. J. Cao H. Jia F. B. Liu X. Q. Shi G. L. (2000). “In Entomology in China in 21st century,” in *Proceedings of Conference of Chinese Entomological Society*, in *Survey of Pesticidal Component in Plant*, ed. Dianmo L. (Beijing: Science & Technique Press), 1098–1104.

[B62] Liu Y. Q. Zhao Y. L. Yang L. Zhou X. W. Feng G. (2012). Design, semisynthesis and insecticidal activity of novel podophyllotoxin derivatives against *Brontispa longissima* in vivo. *Pest. Biochem. Physiol.* 102 11–18. 10.1016/j.pestbp.2011.09.012

[B63] Lowe D. A. (1992). “Fungal enzymes,” in *Handbook of Applied Mycology*: *Fungal Biotechnology* Vol. 4 eds Arora D. K. Elander R. P. Murekji K. G. (New York, NY: Marcel Dekker Inc), 681–706.

[B64] Lubzens E. Minkoff G. Marom S. (1985). Salinity dependence of sexual and asexual reproduction in the rotifer *Brachionus plicatilis*. *Mar. Biol.* 85 123–126. 10.1007/BF00397430

[B65] Luz C. Tai M. H. Santos A. H. Rocha L. F. Albernaz D. A. Silva H. H. (2007). Ovicidal activity of entomopathogenic hyphomycetes on *Aedes aegypti* (Diptera: Culicidae) under laboratory conditions. *J. Med. Entomol.* 44 799–804. 10.1093/jmedent/44.5.799 17915511

[B66] Lv A. Li Z. L. Du F. S. Li Z. C. (2014). Synthesis, functionalization, and controlled degradation of high molecular weight polyester from itaconic acid via ADMET polymerization. *Macromolecules* 47 7707–7716. 10.1021/ma5020066

[B67] Maketon M. Amnuaykanjanasin A. Kaysorngup A. (2014). A rapid knockdown effect of *Penicillium citrinum* for control of the mosquito *Culex quinquefasciatus* in Thailand. *World J. Microbiol. Biotechnol.* 30 727–736. 10.1007/s11274-013-1500-4 24078109

[B68] Maurya P. Mohan L. Sharma P. Srivastava C. N. (2011). Evaluation of larvicidal potential of certain insect pathogenic fungi extracts against *Anopheles stephensi* and *Culex quinquefasciatus*. *Entomol. Res.* 41 211–215. 10.1111/j.1748-5967.2011.00347.x

[B69] Meyer B. N. Ferrigni N. R. Putnam J. E. Jacobsen L. B. Nichols D. J. McLaughlin J. L. (1982). Brine shrimp: a convenient general bioassay for active plant constituents. *Planta Med.* 45 31–34. 10.1055/s-2007-971236 7100305

[B70] Miao F. P. Li X. D. Liu X. H. Cichewicz R. H. Ji N. Y. (2012). Secondary metabolites from an algicolous *Aspergillus versicolor* strain. *Mar. Drugs* 10 131–139. 10.3390/md10010131 22363226 PMC3280527

[B71] Misato T. (1983). “Recent status and future aspects of agricultural antibiotics,” in *Natural Products Pesticide Chemistry: Human Welfare and the Environment* Vol. 2 eds Miyamoto J. Kearney P. C. (Oxford: Pergamon Press), 241–246.

[B72] Mohanty S. S. Kaamaraju R. Usha R. Aditiya P. D. (2008). Efficacy of female *Culex quinquefasciatus* with entomopathogenic fungus *Fusarium pallidoroseum*. *Parasitol. Res.* 103 171–174. 18327611 10.1007/s00436-008-0946-z

[B73] Mohanty S. S. Prakash S. (2008). Laboratory and field evaluation of the fungus *Chrysosporium lobatum* against the larvae of the mosquito Culex quinquefasciatus. *Parasitol. Res.* 102 881–886. 10.1007/s00436-007-0843-x 18193456

[B74] Moretti A. Mule G. Ritieni A. Logrieco A. (2007). Further data on the production of beauvericin, enniatins and fusaproliferin and toxicity to *Artemia salina* by *Fiusarium* species of *Gibberella fujikuroi* species complex. *Int. J. Food Microbiol.* 118 158–163. 10.1016/j.ijfoodmicro.2007.07.004 17706820

[B75] Muema J. M. Njeru S. N. Colombier C. Marubu R. M. (2016). Methanolic extract of *Agerantum conyzoides* exhibited toxicity and growth disruption activities against *Anopheles gambiae* sensu stricto and *Anopheles arabiensis* larvae. *BMC Complement. Alternat. Med.* 16:475. 10.1186/s12906-016-1464-7 27876055 PMC5120420

[B76] Nguta M. J. M Mbaria J. W Gakuya D. K Gathumbi P. D Kabasa J. G Kiama S. (2012). Evaluation of acute toxicity of crude plant extracts from kenyan biodi-versity using brine shrimp, Artemia salina l.(artemiidae). *Open Conference Proc. J.* 3 30–34.

[B77] Nunes B. S. Carvalho F. D. Guilhermino L. M. Van Stappenn G. (2006). Use of the genus *Artemia* in ecotoxicity testing. *Environ. Pollut.* 144 453–462. 10.2174/2210289201203010030 16677747

[B78] Osman M. E. Khatta O. H. Elsab Y. M. (2014). *Aspergillus terreus* proteases: characterization and applications. *J. Chem. Biol. Phys. Sci.* 4 2333–2346. 10.1016/j.envpol.2005.12.037 16677747

[B79] O’Donnell K. Kistler H. C. Cigelnik E. Ploetz R. C. (1998). Multiple evolutionary origins of the fungus causing Panama disease of banana: concordant evidence from nuclear and mitochondrial gene genealogies. *Proc. Natl. Acad. Sci. U.S.A.* 95 2044–2049. 10.1073/pnas.95.5.2044 9482835 PMC19243

[B80] Persoone G. Wells P. G. (1987). “Artemia in aquatic toxicology: a review,” in *Artemia research and its application (Morphology, genetics, strain characterization, Vol. 1)*. Sorgeloos P. Bengtson D. A. Decleir W. Jasper F. (Wetteren: Toxicology – Universa Press), 259–275.

[B81] Passone M. A. Rosso L. C. Ciancio A. Etcheverry M. (2010). Detection and quantification of *Aspergillus* section *Flavi* spp. in stored peanuts by real-time PCR of nor-1 gene, and effects of storage conditions on aflatoxin production. *Int. J. Food Microbiol.* 138 276–281. 10.1016/j.ijfoodmicro.2010.01.003 20153541

[B82] Pastor F. J. Guarro J. (2014). Treatment of *Aspergillus terreus* infections: a clinical problem not yet resolved. *Int. J. Antimicrob. Agents* 44 281–289. 10.1016/j.ijantimicag.2014.07.002 25190543

[B83] Patil C. D. Borase H. P. Patil S. V. Salunkhe R. B. Salunke B. K. (2012). Larvicidal activity of silver nanoparticles synthesized using *Pergularia daemia* plant latex against *Aedes aegypti* and *Anopheles stephensi* and nontarget fish *Poecillia reticulata*. *Parasitol. Res.* 111 555–562. 10.1007/s00436-012-2867-0 22371271

[B84] Perumalsamy H. Chang K. S. Park C. Ahn Y. J. (2013). Larvicidal activity of *Asarum heterotropoides* root constituent’s against insecticide susceptible and resistant *Culex pipiens* pallens and *Aedes aegypti* and *Ochlerotatus togoi*. *J. Agric. Food Chem.* 58 10001–10006. 10.1021/jf102193k 20806890

[B85] Planche A. Kleandrova V. V. Scotti M. T. (2012). Fragment based approach for the in silico discovery of multi-target insecticides. *Chemometr. Intell. Lab. Syst.* 111 39–45. 10.1016/j.chemolab.2011.11.010

[B86] Radhika Rajasree S. R. Ganesh Kumar V. Stanley Abraham L. Indabakandan D. (2010). Studies on the toxicological effects of engineered nanoparticles in environment—a review. *Int. J. Appl. Bioeng.* 4 44–53. 10.18000/ijabeg.10070

[B87] Ragavendran C. Mariappan T. Natarajan D. (2017). Larvicidal, histopathological efficacy of *Penicillium daleae* against larvae of *Culex quinquefasciatus* and *Aedes aegypti* plus biotoxicity on *Artemia nauplii* a non-target aquatic organism. *Front. Pharmacol.* 8:773. 10.3389/fphar.2017.00773 29163159 PMC5663693

[B88] Ragavendran C. Natarajan D. (2015). Insecticidal potency of *Aspergillus terreus* against larvae and pupae of three mosquito species *Anopheles stephensi, Culex quinquefasciatu*s, and *Aedes aegypti*. *Environ. Sci. Pollut. Res.* 22 17224–17237. 10.1007/s11356-015-4961-1 26139412

[B89] Raghavendra K. Verma V. Srivastava H. C. Gunasekaran K. Sreehari U. Dash A. P. (2010). Persistence of DDT, malathion & deltamethrin resistance in *Anopheles culicifacies* after their sequential withdrawal from indoor residual spraying in Surat district. *Indian J. Med. Res.* 132 260–264. 20847371

[B90] Ranson H. Hemingway J. (2005) “Glutathione transferases,” in *Comprehensive Molecular Insect Science – Pharmacology* Vol. 5 eds Gilbert L. I. Iatrou K. Gill S. S. (Oxford: Elsevier), 383–402. 10.1016/B0-44-451924-6/00074-0

[B91] Ravindranath G. Kapadnis B. P. (1991). Isolation and extraction of trichodermin from *Fusarium pallidoroseum*, a fungal pathogen of *Anopheles stephensi*. *Ind. J. Microbiol.* 31 267–269.

[B92] Rizna T. D. Minarti. Akhmad D. Hanny M. (2008). “Emodin, an anthraquinone from ethyl acetate extract of *Aspergillus terreus* koji,” in *Proceeding of the International Seminar on Chemistry*, 731–734.

[B93] Rodrigues P. Soares C. Kozakiewicz Z. Paterson R. R. M. Lima N. Venâncio A. (2007). “Identification and characterization of *Aspergillus flavus* and aflatoxins communicating current research and educational topics and trends in applied microbiology,” in *Communicating Current Research and Educational Topics and Trends in Applied Microbiology*, ed. Mendez-Vilas A. (Badajoz: Formatex), 527–534.

[B94] Ross M. K. Streit T. M. Herring K. L. (2010). Carboxylesterases: dual roles in lipid and pesticide metabolism. *J. Pest. Sci.* 35 257–264. 10.1584/jpestics.R10-07 25018661 PMC4087164

[B95] Ruhul Amin A. R. M. Jabbar A. Rashid M. A. (2003). Antibacterial and cytotoxic activities of metabolites isolated from a *Penicillium* strain. *Pak. J. Biol. Sci.* 6 1365–1367. 10.3923/pjbs.2003.1365.1367

[B96] Saad A. E. Khalil M. T. Ragab F. M. Mekawey A. A. El-Wareth M. T. (2014). Efficacy of the fungi *Aspergillus terreus* and *Penicillium janthinellum* as biological control agents against *Biomphalaria alexandrina* snails. *Int. J. Environ. Sci. Eng.* 5 25–37.

[B97] Saghai-Maroof M. A. Soliman K. M. Jorgensen R. A. Allard R. W. (1984). Ribosomal DNA spacer-length polymorphisms in barley: mendelian inheritance, chromosomal location, and population dynamics. *Proc. Natl. Acad. Sci. U.S.A.* 81 8014–8018. 10.1073/pnas.81.24.8014 6096873 PMC392284

[B98] Saini H. K. Sharma R. M. Bami H. L. Sidhu K. S. (1986). Preliminary study on constituents of mosquito coil smoke. *Pesticides* 20 15–18.

[B99] Saitou N. Nei M. (1987). The neighbor-joining method: a new method for reconstructing phylogenetic trees. *Mol. Biol. Evolut.* 4 406–425. 10.1093/oxfordjournals.molbev.a040454 3447015

[B100] Sambrook J. Fritsch E. F. Maniatis T. (1989). *Molecular Cloning: A Laboratory Manual*, 2nd Edn. Plainview, NY: Cold Spring Harbor Laboratory Press.

[B101] Satoh T. Hosokawa M. (2006). Structure, function and regulation of carboxylesterases. *Chem. Biol. Interact.* 162 195–211. 10.1016/j.cbi.2006.07.001 16919614

[B102] Saxena R. C. Jayashree S. Padma S. Dixit O. P. (1994). Evaluation of growth disrupting activity of ageratum-conyzoides crude extract on *Culex quinquefasciatus* (diptera, culicidae). *J. Environ. Biol.* 15 67–74.

[B103] Schimmel T. G. Coffman A. D. Parsons S. J. (1998). Effect of butyrolactone I on the producing fungus, *Aspergillus terreus*. *Appl. Environ. Microbiol.* 64 3707–3712. 9758788 10.1128/aem.64.10.3707-3712.1998 PMC106526

[B104] Seetharaman P. Gnanasekar S. Chandrasekaran R. Chandrakasan G. Syed A. Hodhod M. S. (2017). Isolation of limonoid compound (Hamisonine) from endophytic fungi *Penicillium oxalicum* LA-1 (KX622790) of *Limonia acidissima* L. for its larvicidal efficacy against LF vector, *Culex quinquefasciatus* (Diptera: Culicidae). *Environ. Sci. Pollut. Res.* 24 21272–21282. 10.1007/s11356-017-9770-2 28741206

[B105] Seiber J. N. Coats J. Duke S. O. Gross A. D. (2014). Biopesticides: state of the art and future opportunities. *J. Agric. Food Chem.* 62 11613–11619. 10.1021/jf504252n 25406111

[B106] Senthil-Nathan S. (2007). The use of Eucalyptus leaf exract as a natural larvicidal agent against malarial vector *Anopheles stephensi* Liston (Diptera: culicidae). *Bioresour. Technol.* 98 1856–1860. 10.1016/j.biortech.2006.07.044 16997545

[B107] Senthil-Nathan S. Choi M. Y. Paik C. H. Seo H. Y. (2007). Food consumption, utilization, and detoxification enzyme activity of the rice leaffolder larvae after treatment with *Dysoxylum triterpenes*. *Pest. Biochem. Physiol.* 88 260–267. 10.1016/j.pestbp.2006.12.004

[B108] Senthil-Nathan S. Choi M. Y. Paik C. H. Seo H. Y. Kalivani K. Kim J. D. (2008). Effect of azadirachtin on acetylcholinesterase (AChE) activity and histology of the brown planthopper *Nilaparvata lugens* (Stal). *Ecotoxicol. Environ. Saf.* 70 244–250. 10.1016/j.ecoenv.2007.07.005 17765967

[B109] Sharma A. Kumar S. Tripathi P. (2015). Impact of *Achyranthes aspera* leaf and stem extracts on the survival, morphology and behaviour of an Indian strain of dengue vector, *Aedes aegypti* L. (Diptera: Culicidae). *J. Mosquito Res.* 5 1–9. 10.5376/jmr.2015.05.0007

[B110] Singha S. Adhikari U. Chandra G. (2011). Smoke repellency and mosquito larvicidal potentiality of *Mesua ferra* L. leaf extract against filarial vector *Culex quinquefasciatus* say, Asian Pacific. *J. Trop. Biomed.* 1 S119–S123. 10.1016/S2221-1691(11)60137-8

[B111] Snow R. W. Guerra C. A. Noor A. M. Myint H. Y. Hay S. I. (2005). The global distribution of clinical episodes of *Plasmodium falciparum* malaria. *Nature* 434:214. 10.1038/nature03342 15759000 PMC3128492

[B112] Soonwera M. Phasomkusolsil S. (2016). Effect of *Cymbopogon citratus* (lemongrass) and *Syzygium aromaticum* (clove) oils on the morphology and mortality of *Aedes aegypti* and *Anopheles dirus* larvae. *Parasitol. Res.* 115 1691–1703. 10.1007/s00436-016-4910-z 26796022

[B113] Su T. Mulla M. S. (1998). Ovicidal activity of neem products (azadirachtin) against *Culex tarsalis* and *Culex quinquefasciatus* (Diptera: Culicidae). *J. Am. Mosq. Contr. Assoc.* 14 204–209. 9673924

[B114] Sugumar S. Clarke S. K. Nirmala M. J. Tyagi B. K. Mukherjee A. Chandrasekaran N. (2014). Nanoemulsion of eucalyptus oil and its larvicidal activity against *Culex quinquefasciatus*. *Bull. Entomol. Res.* 104 393–402. 10.1017/S0007485313000710 24401169

[B115] Suresh U. Murugan K. Benelli G. Nicoletti M. Barnard D. R. Panneerselvam C. (2015). Tackling the growing threat of dengue: *Phyllanthus niruri*-mediated synthesis of silver nanoparticles and their mosquitocidal properties against the dengue vector *Aedes aegypti* (Diptera: Culicidae). *Parasitol*. *Res.* 114 1551–1562. 10.1007/s00436-015-4339-9 25669140

[B116] Teixeria M. F. S. Martins M. S. Silva J. C. D. Kirsch L. S. Fernandes O. C. Carneirol A. L. B. (2012). Amazion biodiversity: pigments from *Aspergillus* and *Penicillium*-characterizations, antibacterial activities and their toxicities. *Curr. Trends Biotechnol. Pharm.* 6 300–311.

[B117] Thompson J. D. Gibson T. J. Plewniak F. Jeanmougin F. Higgins D. G. (1997). The CLUSTAL_X windows interface: flexible strategies for multiple sequence alignment aided by quality analysis tools. *Nucl. Acids Res.* 25 4876–4882. 10.1093/nar/25.24.4876 9396791 PMC147148

[B118] Thompson J. D. Higgins D. G. Gibson T. J. (1994). CLUSTAL W: improving the sensitivity of progressive multiple sequence alignment through sequence weighting, position-specific gap penalties and weight matrix choice. *Nucl Acids Res.* 22 4673–4680. 10.1093/nar/22.22.4673 7984417 PMC308517

[B119] Vadivalagan C. Pushparaj K. Murugan K. Panneerselvam C. Del Serrone P. Benelli G. (2017). Exploring genetic variation in haplotypes of the filariasis vector *Culex quinquefasciatus* (Diptera: Culicidae) through DNA barcoding. *Acta Trop.* 169 43–50. 10.1016/j.actatropica.2017.01.020 28126462

[B120] Van Asperen K. (1962). A study of housefly esterases by means of a sensitive colorimetric method. *J. Insect. Physiol.* 8 401–416. 10.1016/0022-1910(62)90074-4

[B121] van der Straat L. Vernooij M. Lammers M. van den Berg W. Schonewille T. Cordewener J. (2014). Expression of the *Aspergillus terreus* itaconic acid biosynthesis cluster in *Aspergillus niger*. *Microb. Cell Fact.* 13:11. 10.1186/1475-2859-13-11 24438100 PMC3898256

[B122] Vatsyayan P. Goswami P. (2016). Highly active and stable large catalase isolated from a hydrocarbon degrading *Aspergillus terreus* MTCC 6324. *Enzyme Res.* 4379403 1–8. 10.1155/2016/4379403 27057351 PMC4807065

[B123] Venkateswara Rao J. Kavitha P. Jakka N. M. Sridhar V. Usman P. K. (2007). Toxicity of organoposphates on morphology and locomotor behavior in brine shrimp, *Artemia salina*. *Arch. Environ. Contam. Toxicol.* 53 227–232. 10.1007/s00244-006-0226-9 17549541

[B124] Vertesy L. Burger H. J. Kenja J. Knauf M. Kogler H. Paulus E. R. (2000). Kodaistatins, novel inhibitors of glucose-6-phosphate translocase T1 from *Aspergillus terreus* thorn DSM 11247. *J. Antibiot.* 53 677–686. 10.7164/antibiotics.53.677 10994809

[B125] Vyas N. Dua K. K. Prakash S. (2007). Efficacy of *Lagenidium giganteum* metabolites on mosquito larvae with reference to non-target organisms. *Parasitol. Res.* 101 385–390. 10.1007/s00436-007-0496-9 17334944

[B126] Waliwitiya R. Nicholson R. A. Kennedy C. J. Lowenberger C. A. (2012). The synergistic effects of insecticidal essential oils and piperonyl butoxide on biotransformational enzyme activities in *Aedes aegypti* (Diptera: Culicidae). *J. Med. Entomol.* 49 614–623. 10.1603/ME10272 22679869

[B127] Waqas M. Khan A. L. Hamayun M. Shahzad R. Kang S.-M. Kim J.-G. (2015). Endophytic fungi promote plant growth and mitigate the adverse effects of stem rot: an example of *Penicillium citrinum* and *Aspergillus terreus*. *J. Plant Interact.* 10 280–287. 10.1080/17429145.2015.1079743

[B128] Warikoo R. Kumar S. (2013). Impact of *Argemone mexicana* extracts on the cidal, morphological, and behavioral response of dengue vector, *Aedes aegypti* L.(Diptera: Culicidae). *Parasitol. Res.* 112 3477–3484. 10.1007/s00436-013-3528-7 23835923

[B129] Wheelock C. E. Phillips B. M. Anderson B. S. Miller J. L. Miller M. J. Hammock B. D. (2008). Applications of carboxylesterase activity in environmental monitoring and toxicity identification evaluations (TIEs). *Rev. Environ. Contam. Toxicol.* 195 117–178. 10.1007/978-0-387-77030-7_5 18418956

[B130] WHO (2010). *Dengue Transmission Research in WHO Bulletin.* Geneva: WHO.

[B131] WHO (2011). *Dengue and Dengue Haemorrhaghic Fever. Fact Sheet 117.* Geneva: WHO.

[B132] WHO (1981). *Instructions for Determining the Susceptibility or Resistance of Adult Mosquitoes to Organochlorine, Organophosphate and Carbamate Insecticides: Diagnostic Test.* Geneva: WHO; WHO/VBC/81.807.

[B133] WHO (2000). Effect of breastfeeding on infant and child mortality due to infectious diseases in less developed countries: a pooled analysis. Collaborative Study Team on the role of breastfeeding on the prevention of infant mortality. *Lancet* 355 451–455. 10.1016/S0140-6736(00)82011-5 10841125

[B134] Yin Y. Cai M. Zhou X. Li Z. Zhang Y. (2016). Polyketides in *Aspergillus terreus*: biosynthesis pathway discovery and application. *Appl. Microbiol. Biotechnol.* 100 7787–7798. 10.1007/s00253-016-7733-z 27455860

