# Supplementary material for: Aspergillus terreus (Trichocomaceae): A Natural, Eco-Friendly Mycoinsecticide for Control of Malaria, Filariasis, Dengue Vectors and Its Toxicity Assessment Against an Aquatic Model Organism Artemia nauplii
Source: Front Pharmacol. 2018 Nov 26;9:1355. doi: 10.3389/fphar.2018.01355 (PMC6275207; doi:10.3389/fphar.2018.01355)
Supplement: Supplementary file 1 [file Data_Sheet_1.doc]

**Supplementary materials**

**S-Figure** **1.** Phylogenetic tree construction of the *A. terreus* (based on ITS1, 5.8S and ITS 2 regions sequences, where evolutionary history was inferred by the Neighbour-joining method. Bootstrap values >50% (1000 replicates) are revealed the branches. The bar indicates a 5% sequence divergence. The analysis totally involved 22 nucleotide sequences and 420 positions in the final dataset. Evolutionary analysis was conducted using MEGA 6).

**S-Figure 2.** Neurobehavioral toxicity indications in the 4^th^ instar larvae of *Ae. aegypti*, influenced after the treatment by the ethyl acetate metabolite of *A. terreus*: **a**) larvae from metabolite treatment cluster showing normal behaviours, **b**) control larvae showing normal behaviours, **c**) mycelia extract treated larvae group displayed weakened coordination (I), movements changed (II), and forceful self-biting behaviours (III) (200µg/ml), **d**) control larvae group exhibited regular behaviours, **e**) extract treated larvae group displayed movements were vibrating and lack of sensation sign (200µg/ml) and **f**) control larvae group exhibited natural sign, NB, normal behaviours, P, paralysis; T, tremors; RS, respiratory siphon.

**S-Figure 3.** Growth disruption effects mediated by *A. terreus* mycelium ethyl acetate extract at 72 h post exposure. **a)** *An. stephensi* control larva. **b)** Abnormal *An. stephensi* larval-pupal intermediate. **c)** Disfigure pupa (DP). Pupa showing manifestation of an elephant and was shaped as “elephantoid”. **d & e**) Disfigure pupa (DP). Pupa showed dwarf pupa with retarded stomach and wing pads are (WP) not appressed to the body. **f)** Aborted adult emergence in *An. stephensi* with legs stuck in pupal caste (Larval morphological appearances were viewed/photographed using light microscopy at 40x magnification *viz*: before and after treatment by mycelia extract).

**S-Figure 4.** Morphogenetic abnormalities of *Cx. quinquefasciatus* induced by *A. terreus* mycelium ethyl acetate extract: **a)** normal fourth instar larva, **b)** growth/molting collapsed larviform pupae (larval-pupal intermediates) **c & d)** after 72 hour of treatment pupa were physically deformed, **e)** Disfigure pupa (DP) with alteration of digestive region and inflamed cephalothorax (DP) **f)** morphogenetic irregular as adult adhesive to the pupal case (PA) of *Cx. quinquefasciatus*.

**S-Figure 5.** Morphological malformation and growth/molting uncontrollable indications were observed in *Ae. aegypti* 4^th^ instar larvae, influenced after the treatment (48 h) with ethyl acetate extract of *A. terreus,* at concentration of 100µg/ml: **a**) and **b**) larvae were morphologically disfigure, **c**) control group larva showed a healthy form, **d**) and **e**) growth/molting distort larviform pupae (larval-pupal intermediates), **f**) pupa appearance as morphologically disfigure (200µg/ml), post 72 h of treatment, **g**) control group showed healthy pupa, **h**) mycelium extract treated adult group revealed a slightly deformed, and **i**) in control group observed healthy adult. HE, head; CE, compound eye; RT, respiratory trumpet; FB, feeding brush; RS, respiratory siphon; TH, thorax; AB, abdomen; DT, digestive tract; AP, anal papillae; CT, cephalothorax; RUA, rudiments of appendages; AB, abdomen; P, paddles; Ex, larval exuviae; W, wing.

**
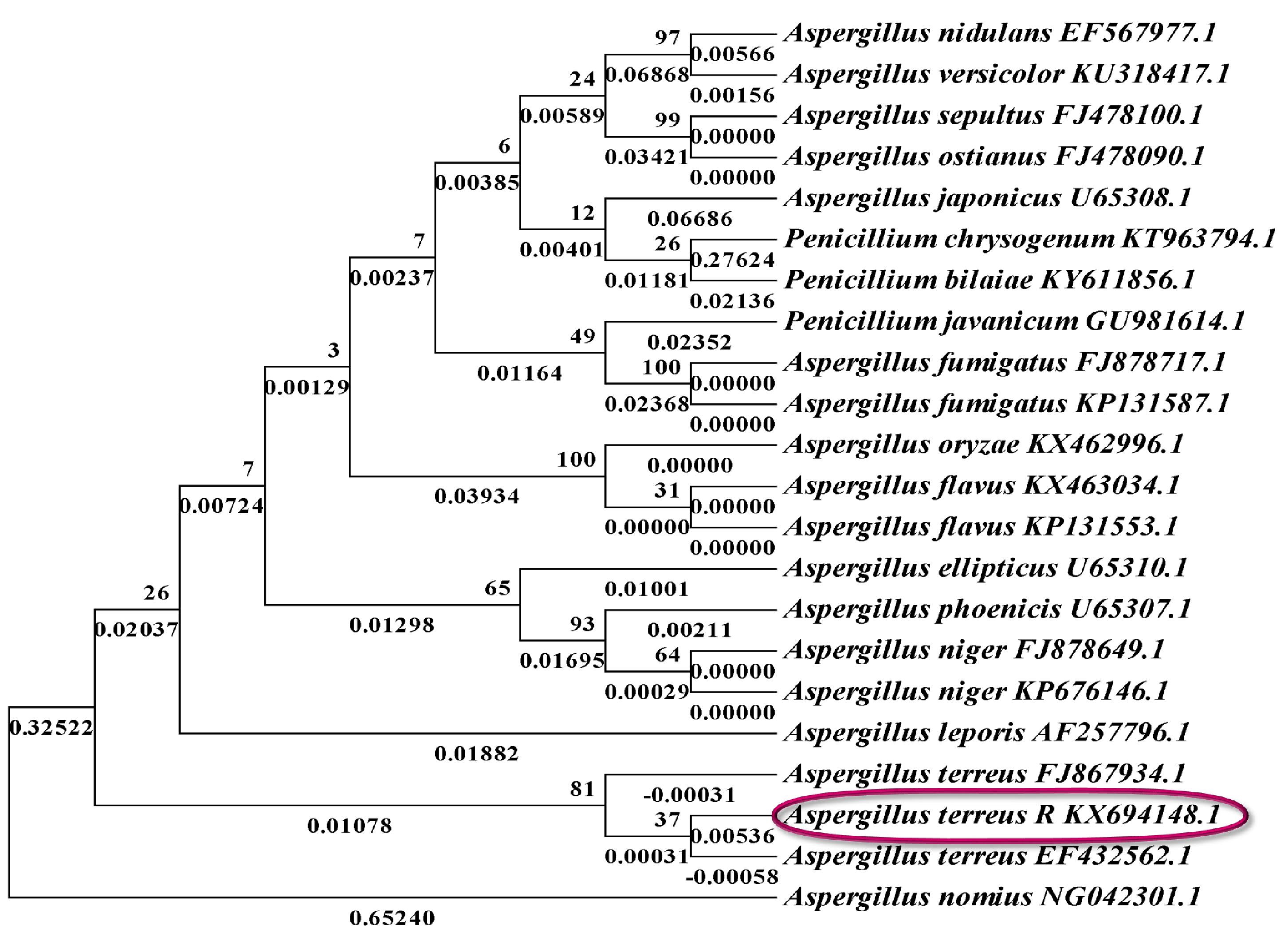
**

**S-Figure** **1.**


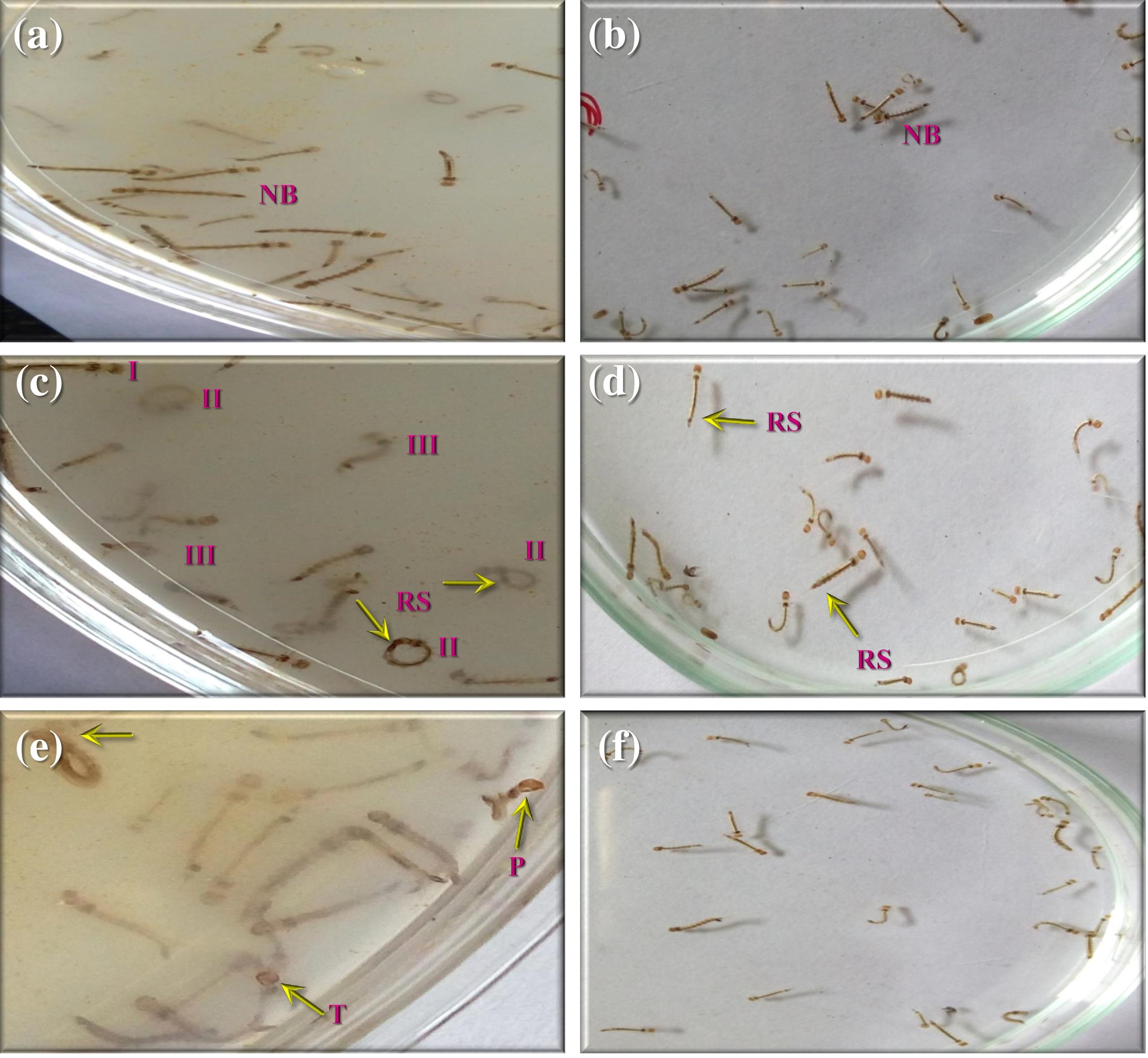


**S-Figure 2.**


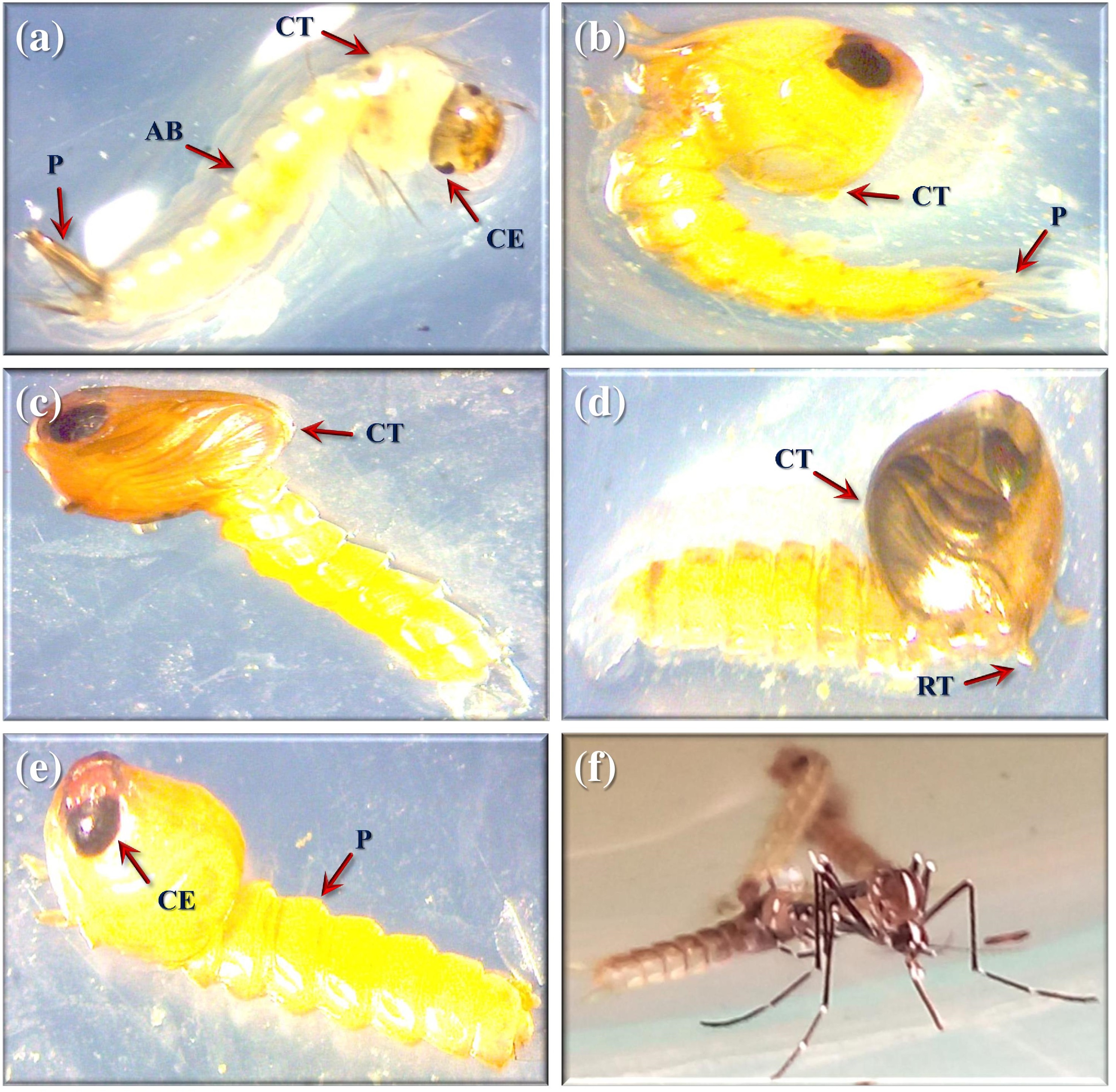


**S-Figure 3.**


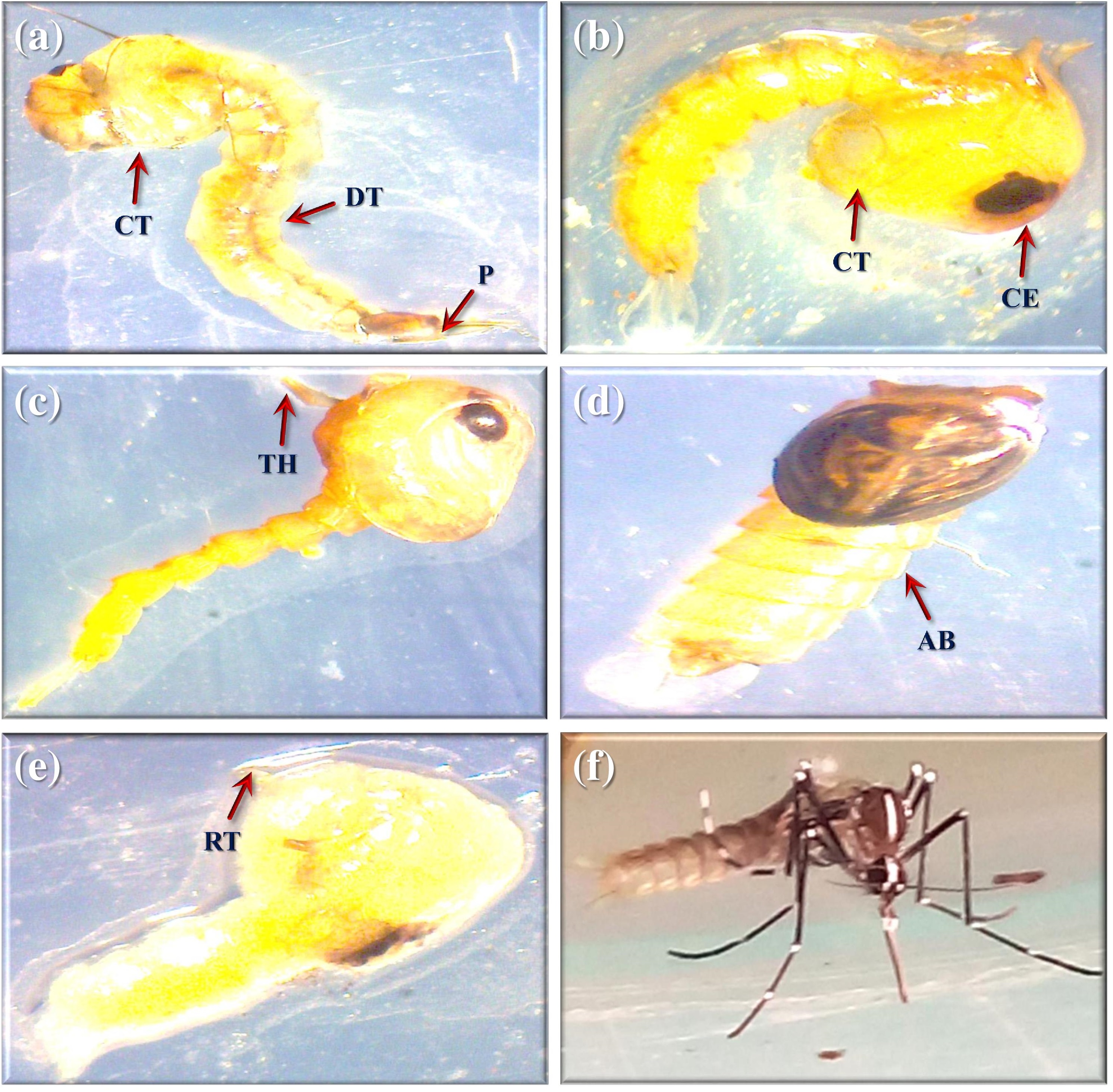


**S-Figure 4.**


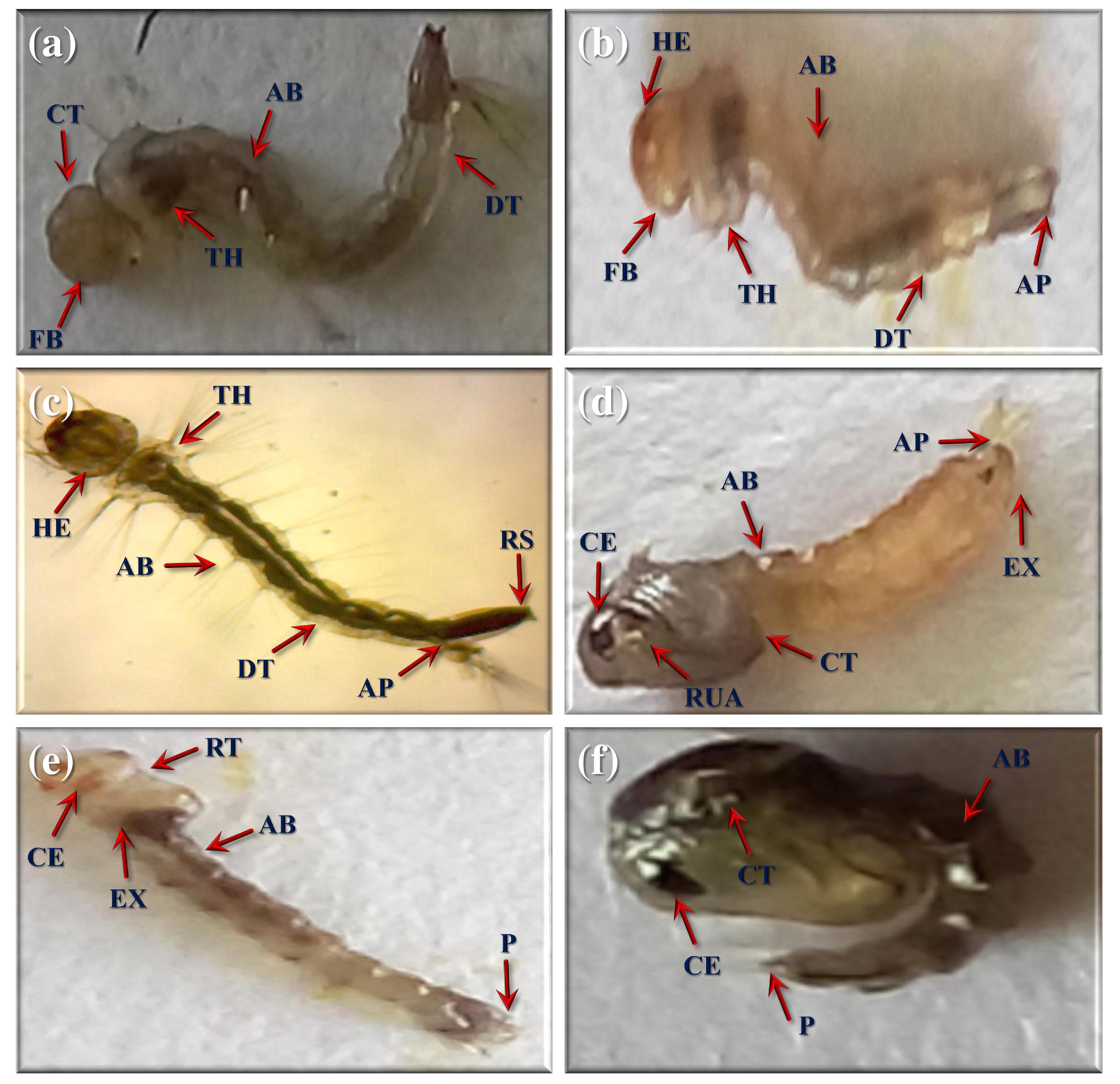


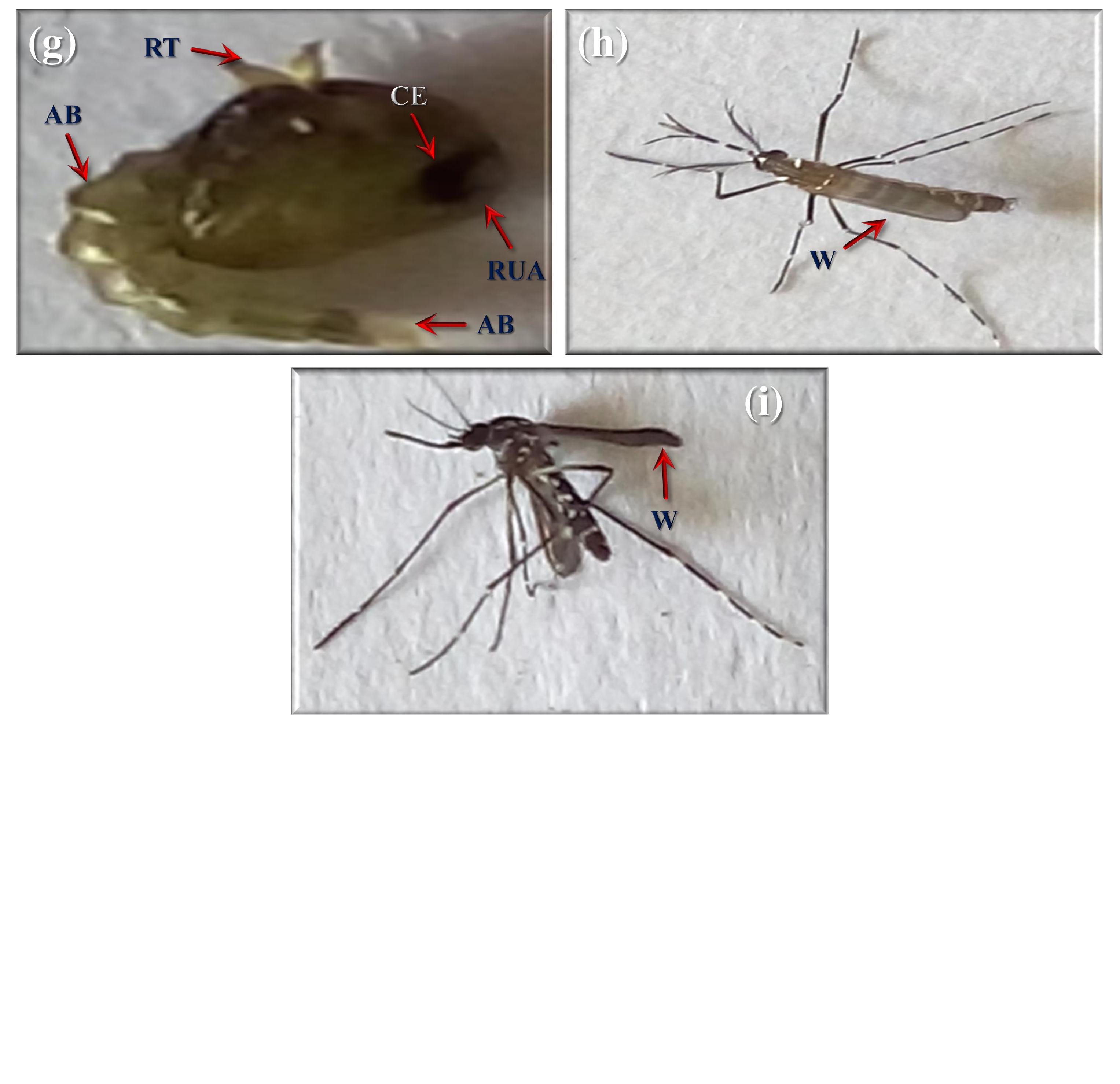


**S-Figure 5.**
